# Discrete multiporphyrin pseudorotaxane assemblies from di- and tetravalent porphyrin building blocks

**DOI:** 10.3762/bjoc.11.85

**Published:** 2015-05-12

**Authors:** Mirko Lohse, Larissa K S von Krbek, Sebastian Radunz, Suresh Moorthy, Christoph A Schalley, Stefan Hecht

**Affiliations:** 1 Department of Chemistry, Humboldt-Universität zu Berlin, Brook-Taylor-Str. 2, 12489 Berlin, Germany. Fax: +49 (0)30 2093-6940; Tel: +49 (0)30 2093-7308; 2 Institut für Chemie und Biochemie, Freie Universität Berlin, Takustraße 3, 14195 Berlin, Germany. Fax: +49(0)308385-5366; Tel: +49(0)308385-2639

**Keywords:** crown ethers, multicomponent assembly, multivalency, porphyrins, pseudorotaxanes

## Abstract

Two pairs of divalent and tetravalent porphyrin building blocks carrying the complementary supramolecular crown ether/secondary ammonium ion binding motif have been synthesized and their derived pseudorotaxanes have been studied by a combination of NMR spectroscopy in solution and ESI mass spectrometry in the gas phase. By simple mixing of the components the formation of discrete dimeric and trimeric (metallo)porphyrin complexes predominates, in accordance to binding stoichiometry, while the amount of alternative structures can be neglected. Our results illustrate the power of multivalency to program the multicomponent self-assembly of specific entities into discrete functional nanostructures.

## Introduction

Supramolecular chemistry [1], the chemistry “beyond the molecule“ [2], has immensely reshaped the concepts of chemistry by putting the intermolecular interaction into the focus. Different fields of chemistry, from materials [3–6] and analytical sciences [7–12] to life science [13–17] have benefited from the development of the basic concepts of molecular recognition, templation [18], self-assembly [19], or self-sorting [20–21], just to name a few. More recently, multivalent binding [22–24] and cooperativity [25–26] have attracted significant attention mediated in particular by the desire to understand biological phenomena, such as virus docking to cells [27], toxin inhibition [28], or leucocyte recruitment in inflammation processes of the endothelium [29]. Multivalency has also inspired synthetic supramolecular architecture as it not only contributes to binding enhancement, but also helps to exert control over complex formation. For example, “molecular elevators” have been constructed by Stoddart et al. [30–31] and giant porphyrin wheels were prepared by Anderson and co-workers [32–33], both using a multivalent template strategy.

The crown ether/secondary ammonium ion binding motif [34] is a powerful tool to create well-defined pseudorotaxane structures [35–39], which have also served as precursors in rotaxane syntheses [40–42] thus providing access to interlocked, mechanically bound molecules. Based on these structures, functional supramolecular architectures such as molecular switches and motors [43–45] as well as artificial muscles [46–50], have been synthesized.

Due to their four-fold symmetry, porphyrins are excellent candidates to extend these concepts to tetravalent supramolecules. Beyond being a mere spacer and scaffold connecting the binding sites, porphyrins also offer interesting physical and optical properties [51–52]. Therefore, they have played a pivotal role in supramolecular chemistry [53–66], for example as potential candidates for artificial light-harvesting systems [67–73].

**Figure 1:**
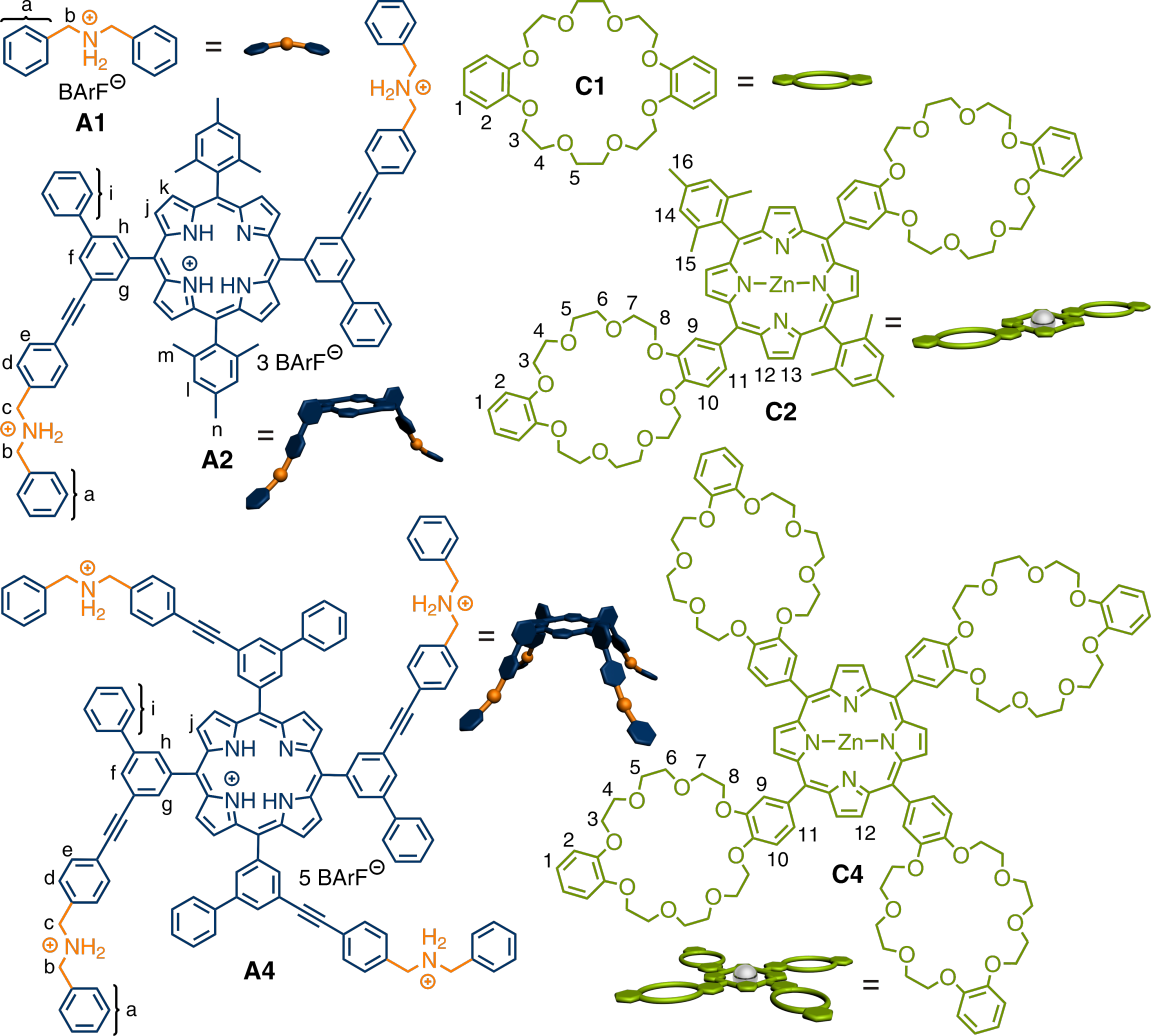
Mono-, di-, and tetravalent axles **A1**, **A2** and **A4** and mono-, di-, and tetravalent hosts **C1**, **C2** and **C4**. Numbers and letters are assigned to specific H atoms as discussed later in the main text.

Here, we report the synthesis of two new porphyrin-based di- and tetravalent ammonium guest molecules **A2** and **A4** and their complementary porphyrin-based di- and tetravalent crown ether hosts **C2** and **C4** (Figure 1). The selection of these building blocks is based on force-field calculations, which suggest a good geometric fit between the crown ether hosts and the ammonium ion guests. The two monovalent building blocks **A1** and **C1** serve as control compounds. Based on this “toolbox”, which can be expanded in the future with other functional building blocks, the formation of specific multiply threaded pseudorotaxanes was achieved, thereby demonstrating the ability to program complex multicomponent self-assembly [74–75].

## Results and Discussion

### Synthesis

**Scheme 1:**
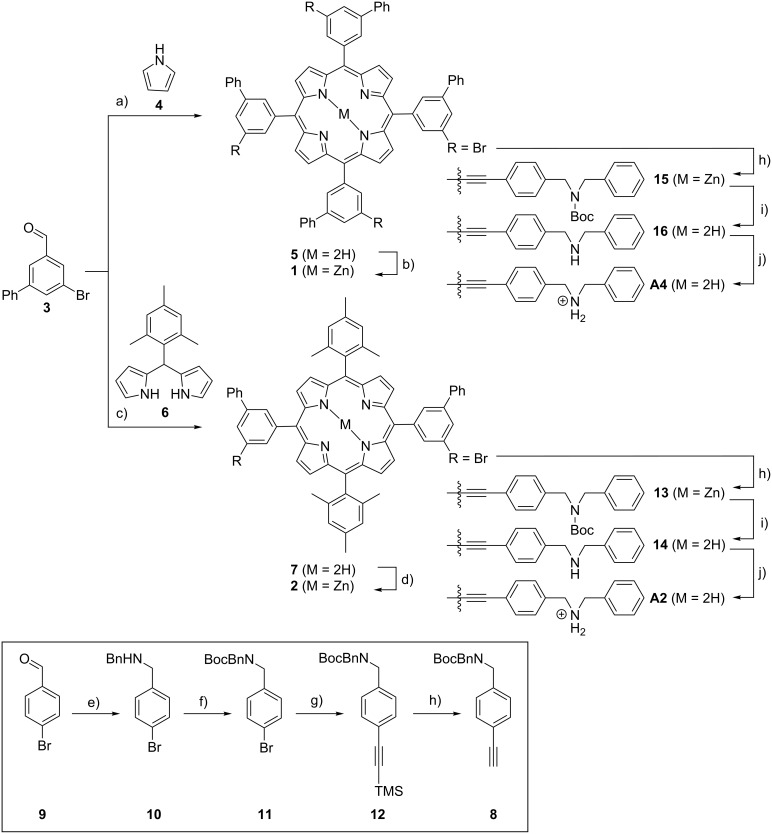
Overview of the synthesis of the guests **A2** and **A4**. a) Pyrrole (**4**), BF_3_·Et_2_O, DDQ, CHCl_3_, rt; b) Zn(OAc)_2_, CHCl_3_/MeOH, rt; c) dipyrromethane **6**, BF_3_·Et_2_O, DDQ, CHCl_3_, rt; d) Zn(OAc)_2_, CHCl_3_/MeOH, rt; e) 1. benzylamine, trimethyl orthoformate, rt, 2. NaBH_4_, THF/MeOH, rt; f) Boc_2_O, triethylamine, CH_2_Cl_2_, rt; g) 1. ethynyltrimethylsilane, CuI, PPh_3_, Pd(PPh_3_)_4_, TEA, toluene, 80 °C, 2. KOH, THF, rt; h) precursor **8**, CuI, PPh_3_, Pd(PPh_3_)_4_, TEA, toluene, 80 °C; i) TFA, CH_2_Cl_2_, rt; j) 1. HCl, MeOH/CHCl_3_, rt, 2. NaBArF, MeOH.

The synthesis of the two ammonium-substituted porphyrins **A2** and **A4** was performed convergent by first preparing two different (zinc)porphyrin cores **1** and **2** (Scheme 1), which are equipped with two and four bromine atoms in the *m*-position of the *meso*-phenyl substituents, respectively, for further functionalization. Zinc porphyrins **1** and **2** have been synthesized following standard protocols for symmetrical [76] A_4_ and *trans*-disubstituted [77] A_2_B_2_ *meso*-functionalized porphyrins. The tetrabrominated core **1** was synthesized from aldehyde **3** and pyrrole (**4**) to form the free base porphyrin **5**, which is subsequently converted into its zinc complex **1**. On the other hand the difunctional core **2** was obtained through the condensation of aldehyde **3** with mesityldipyrromethane (**6**) followed by metalation of the intermediately formed free base porphyrin **7** to give its respective zinc complex **2**. In the next step, axle precursor **8** was synthesized by reductive amination of 4-bromobenzaldehyde (**9**) and benzylamine yielding amine **10**, which was subsequently Boc-protected, then reacted with trimethylsilylacetylene in a Sonogashira cross-coupling followed by desilylation. Finally, the porphyrin cores **1** and **2** were combined with axle precursor **8** in another two and four-fold Sonogashira cross-coupling reaction. After deprotection of the termini of the attached axles with trifluoroacetic acid (TFA), protonation of the free amines with HCl, and anion exchange with sodium tetrakis(3,5-bis(trifluoromethyl)phenyl)borate (NaBArF), the target compounds **A2** and **A4** were obtained. The weakly coordinating BArF counter-ion has been used to overcome solubility problems in organic solvents. It should be noted that the porphyrin is demetalated to yield the free base porphyrin during the deprotection of the Boc group. Furthermore, NMR integration of signals corresponding to the BArF protons relative to those corresponding to the macrocycle indicates that the porphyrin core is protonated (three BArF anions per divalent guest **A2**; five BArF anions per tetravalent guest **A4**). Based on the assumption that protonation of the porphyrin core, which is rather remote to the primary binding sites, does not influence the association strongly, no selective deprotonation of the porphyrin core has been attempted.

**Scheme 2:**
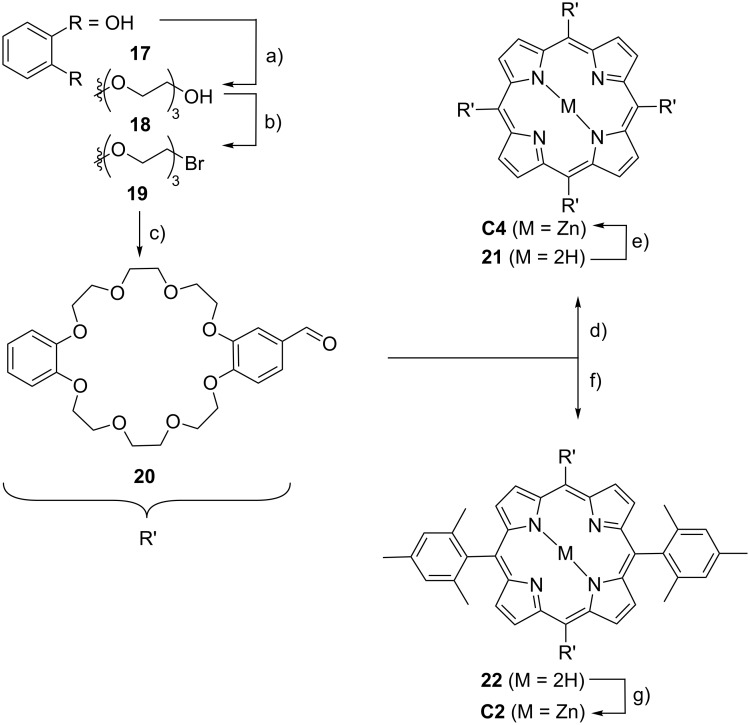
Synthesis of crown ether hosts **C4** and **C2**: a) K_2_CO_3_, LiBr, **17**, 2-[2-(2-chloroethoxy)ethoxy]ethanol, DMF, 100 °C; b) CBr_4_, PPh_3_, CH_2_Cl_2_, rt; c) Cs_2_CO_3_, 3,4-dihydroxybenzaldehyde, DMF, 85 °C; d) 1. pyrrole (**4**), propionic acid, 140 °C, 2. Zn(OAc)_2_, MeOH/CHCl_3_, rt; e) 1. dipyrromethane (**6**), BF_3_·Et_2_O, DDQ, CHCl_3_, rt, 2. Zn(OAC)_2_, MeOH/CHCl_3_, rt.

The preparation of the corresponding crown ether hosts (Scheme 2) involved an initial Williamson ether synthesis in which catechol (**17**) was first extended with 2-[2-(2-chloroethoxy)ethoxy]ethanol to diol **18**, which was then converted in dibromide **19** by an Appel reaction. Macrocyclization of **19** with 3,4-dihydroxybenzaldehyde under “pseudo high-dilution” conditions, i.e., slow addition of the two reactants into a solution of Cs_2_CO_3_ in DMF at 100 °C provides the corresponding crown ether aldehyde **20**. Porphyrin synthesis using **20** and pyrrole (**4**) following the Lindsey protocol [77] for A_4_ porphyrins gives the desired tetravalent porphyrin host as the free base **21**, which is subsequently converted into the desired product **C4** by metalation using zinc(II) acetate. Host **C2** was synthesized according to the above-mentioned standard procedure [76] for *trans*-A_2_B_2_-porphyrins from **20** and mesityldipyrromethane **6** to form the divalent free base porphyrin **22**. Final zinc insertion provides the desired host **C2**.

For further detailed synthetic procedures and characterization data the reader is referred to Supporting Information File 1.

### Formation and characterization of complexes

NMR spectroscopy of simple pseudorotaxanes prepared from crown ether wheels and secondary ammonium axles provides complexation-induced shift data, which can be easily interpreted and yield insight into complexation. Earlier experiences with divalent crown/ammonium pseudorotaxanes however also demonstrated that the NMR spectroscopic approach is often rather limited for more complex structures [78], as very complicated spectra are obtained with typically overlapping signals that prevent further (straightforward) analysis. Another complication, which makes the NMR analysis difficult, is the fact that the di- and tetravalent crown ethers **C2** and **C4** are achiral themselves, but become chiral, when complexed to axle components **A2** and **A4**. Consequently, the signals for all methylene protons of the crown ethers split into two diastereotopic ones not only producing another set of signals, but also more complicated splitting patterns. Furthermore, the crown ethers are connected to the porphyrin core by single bonds, around which they can easily rotate in the non-complexed state. This rotation is, however, fixed upon complexation and two possible orientations of each of the crown ethers on its corresponding axle are possible. One can therefore expect a mixture of stereoisomers to form. In the simplest case, **A2**@**C2**, two enantiomers and one *meso*-form are expected to exist, which should result in two overlapping sets of signals. For the other three complexes, the situation is even more complicated. Therefore, a straightforward and easy analysis of the NMR spectra will likely be impossible.

**Figure 2:**
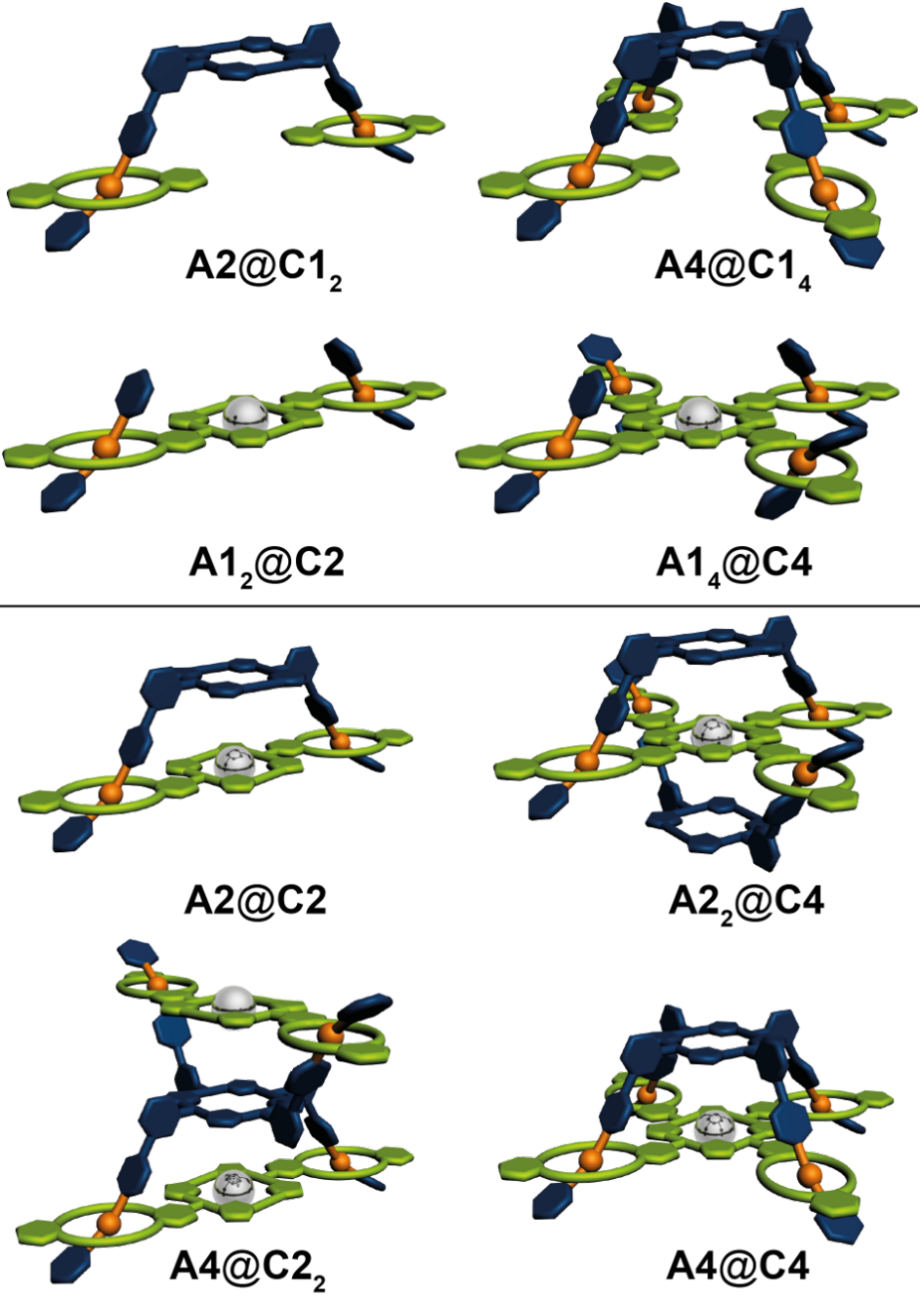
Schematic representation of the host–guests complexes. Top: complexes **A2**@**C1_2_**, **A4**@**C1_4_**, **A1_2_**@**C2** and **A1_4_**@**C4**, which are built from one multi- and several monovalent building blocks. Bottom: complexes **A2**@**C2, A2_2_**@**C4, A4**@**C2_2_** and **A4**@**C4**, which are built from di- or tetravalent building blocks.

In our earlier studies [37,78–79], however, electrospray ionization (ESI) mass spectrometry (MS) turned out to be a perfectly suited method to characterize the complexes present in solution. The formation of unspecific complexes as well as fragmentation upon ionization have been found to be quite limited so that the picture obtained from the mass spectra can be expected to provide realistic insights into the composition of the complexes present in solution. As all stereoisomers have the same elemental composition, their presence as a mixture does not obscure the mass spectrometric results. For these reasons, we describe our NMR spectroscopic data, but focus on ESI–MS of the complexes under study starting with the four possible combinations of **A2** and **A4** with monovalent dibenzo[24]crown-8 **C1** as well as of **C2** and **C4** with monovalent dibenzylammonium **A1** (Figure 2, top), followed by the results obtained for the multivalent 1:1 and 2:1 complexes **A2**@**C2**, **A2_2_**@**C4**, **A4**@**C2_2_** and **A4**@**C4** (Figure 2, bottom).

### [3]- and [5]pseudorotaxanes from monovalent building blocks

**Figure 3:**
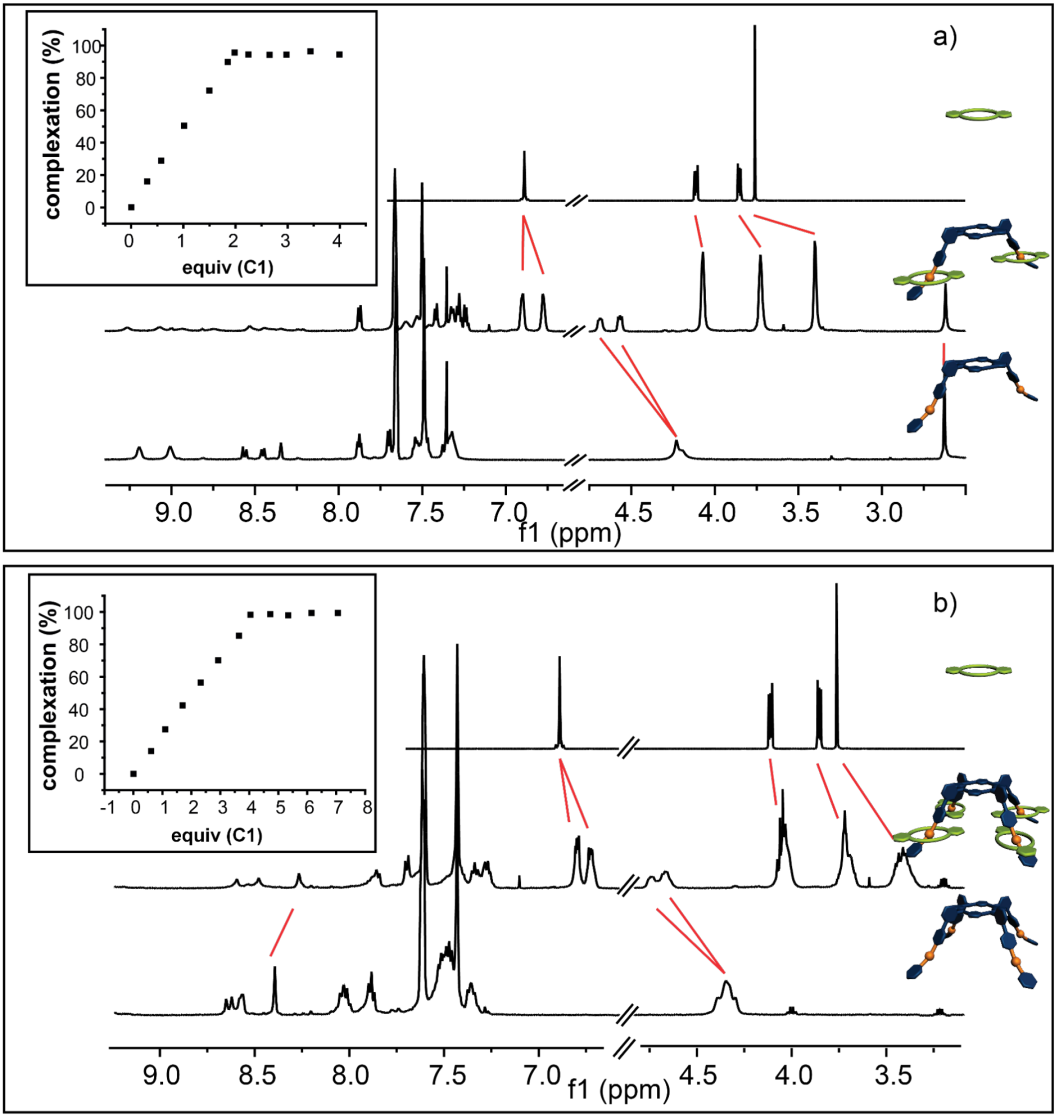
^1^H NMR (500 MHz, 298 K, CD_2_Cl_2_, 3 mM) of a) **C1** (top), **A2**@**C1_2_** (middle) and **A2** (bottom); b) **C1** (top), **A4**@**C1_4_** (middle) and **A4** (bottom) showing clear evidence of the complexation. The red lines indicate the shift of the proton signals upon addition. The inserts show the titration curve of each complexation with the expected ratio of the complex formed.

**Figure 4:**
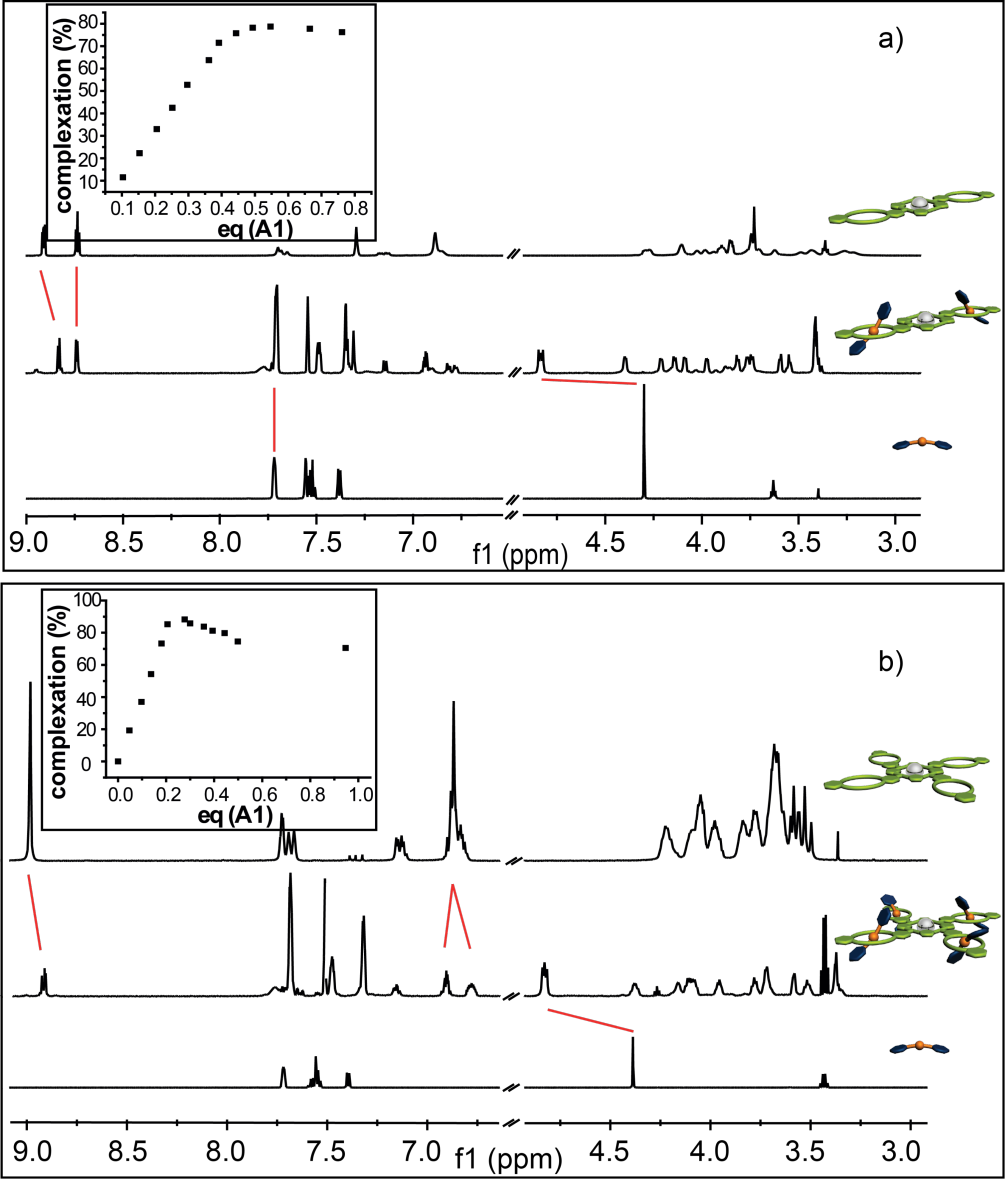
^1^H NMR (500 MHz, 298 K, CD_2_Cl_2_, 3 mM) of a) **C2** (top), **A1_2_**@**C2** (middle) and **A1** (bottom) and b) **C4** (top), **A1_4_**@**C4** (middle) and **A1** (bottom) showing clear evidence of the complexation. The red lines indicate the shift of the proton signals upon addition. The inserts show the titration curve of each complexation with the expected ratio of the complex formed.

First the association of **A2** and **A4** with monovalent **C1** as well as **C2** and **C4** with monovalent **A1** was investigated and it can be seen that in all four cases successful complexation with the expected stoichiometry was achieved. For instance, upon addition of **C1** to a 3 mM solution of **A2** (Figure 3a) a continuous complexation, indicated by the appearance of a new set of signals due to slow exchange rates on the NMR-time scale, could be observed. Upon association the benzyl signals H^b/c^ shift downfield by approximately +0.3 ppm and split into two separate pair of signals, which is typical for a complexation of **C1** with a dibenzylammonium moiety [36]. The aromatic signals of **C1** H^1/2^ shift slightly upfield by −0.1 ppm and split as well. The signals of the crown ether region shift upfield by −0.05, −0.14, and −0.38 ppm due to complexation. An overstoichiometric addition of **C1** results in no further association (see Figure 3a, inset), clearly proving the desired host–guest ratio in the supramolecular structure. Similar results are obtained for the other [3]- and [5]pseudorotaxanes (Figure 3b and Figure 4a,b). However, it should be noted that despite extensive titration experiments (see Supporting Information File 1 for details) a detailed analysis of the binding constants of these systems cannot be obtained as the binding constants are too high for a NMR-based method.

**Figure 5:**
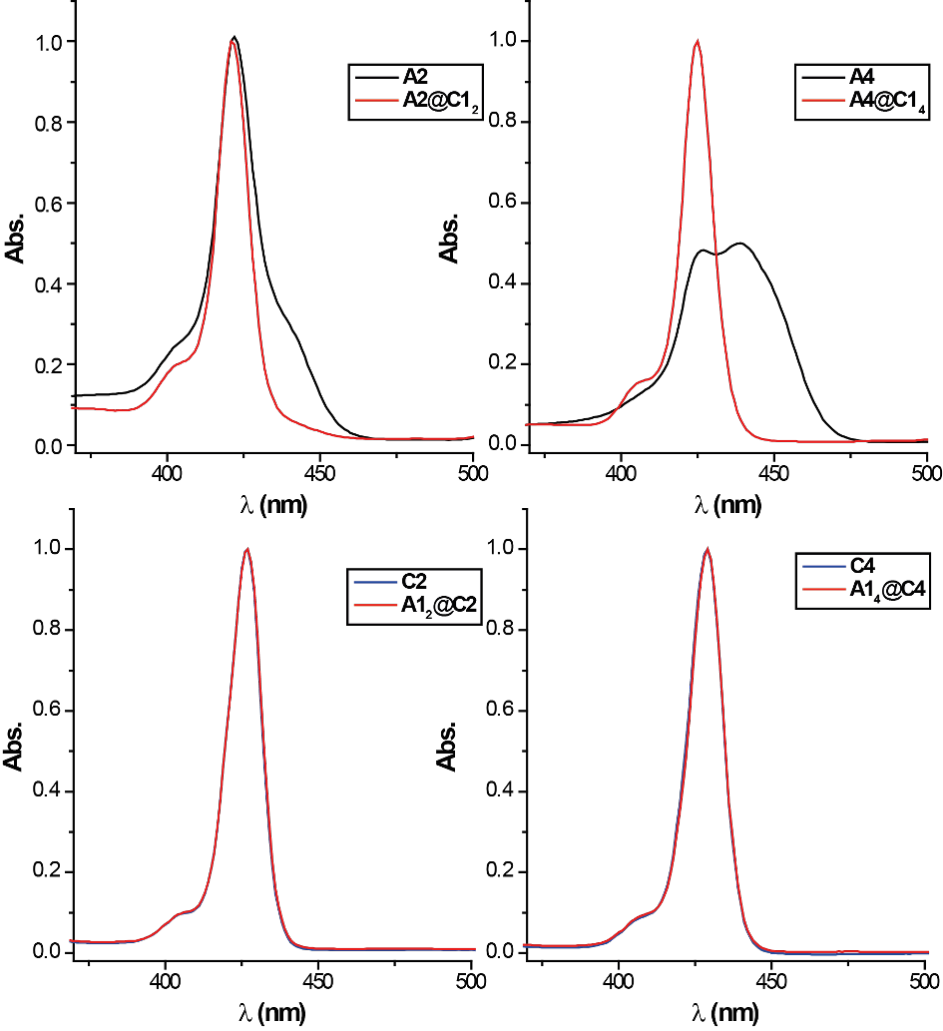
Normalized UV–vis absorption spectra (CH_2_Cl_2_, 3 μM) of **A2**, **A4**, **C2** and **C4** and their complexes formed with the monovalent building blocks **A1** and **C1** showing no significant batho- or hypsochromic shift. Absorption spectrum of **A4** was normalized to 0.5 because of the strong self-aggregation and the resulting broad Soret band.

Guests **A2** and **A4** as well as the hosts **C2** and **C4** show typical absorption behavior for porphyrin-based molecules. All four have pronounced absorption maxima at around 420 nm (Soret band) and less intense absorption bands between 500 and 600 nm (Q-bands). However, **A4** shows rather strong aggregation even in the µM concentration regime likely caused by electrostatic interactions mediated by the closely associated BArF counter-ions that are expected to be significant as rather non-polar solvents are being used. This aggregation results in a broad red-shifted absorption band. Upon complexation this aggregate is broken, resulting in the recovery of a typical sharp Soret band at 420 nm. Note that UV–vis titration shows no significant batho- or hypsochromic shift upon association (Figure 5) of neither di- and tetravalent guests **A2** and **A4** with monovalent host **C1** nor of monovalent guest **A1** to the di- and tetravalent hosts **C2** and **C4**. The lack of such optical signature of the complexation event in the characteristic porphyrin absorption can be explained by the fact that the binding sites are electronically decoupled from the porphyrin core.

**Figure 6:**
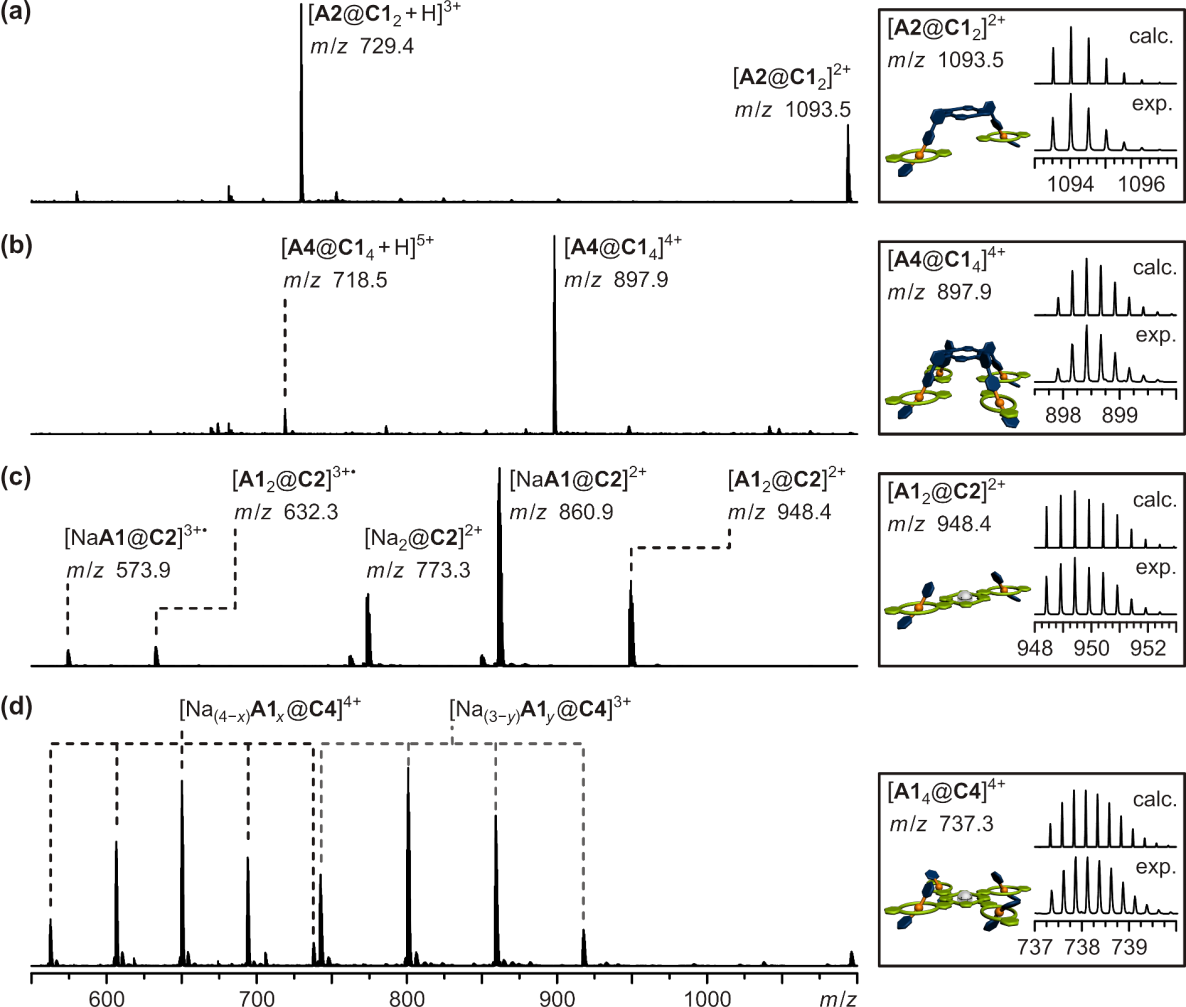
ESI-Q-TOF-MS spectra (CH_2_Cl_2_, 0.2 µM; left hand side) and respective experimental and calculated isotopic patterns of the desired [3]- or [5]pseudorotaxanes (right hand side): a) 1:2 mixture of **A2** and **C1**, b) 1:4 mixture of **A4** and **C1**, c) 2:1 mixture of **A1** and **C2**, d) 4:1 mixture of **A1** and **C4**. For reasons of clarity not all of the peaks are assigned (see Supporting Information File 1 for details).

The [3]- and [5]pseudorotaxanes with the monovalent building blocks were further investigated by ESI-Q-TOF mass spectrometry. Separate solutions of hosts and guests were prepared (**A1**/**C1**: 4 mM, **A2**/**C2**: 2 mM, **A4**/**C4**: 1 mM all in CH_2_Cl_2_), and the same aliquots of the individual solutions were combined to obtain equal concentrations of ammonium ion functions and crown ether moieties in each solution. The solutions of the pseudorotaxanes were allowed to equilibrate for 24 hours at room temperature and diluted to 0.2 µM prior to analysis. The respective [3]- or [5]pseudorotaxanes could be detected for all mixtures (Figure 6). In the cases of the 1:2 and 1:4 mixtures of **A2** and **A4** with **C1**, respectively, the respective pseudorotaxanes **A2**@**C1**_2_ and **A4**@**C1**_4_ give rise to the second and third most abundant species (Figure 6a,b). One signal represents the desired doubly, respectively quadruply charged pseudorotaxane ([**A2**@**C1**_2_]^2+^ at *m*/*z* 1094 and [**A4**@**C1**_4_]^4+^ at *m*/*z* = 898). In addition, a second set of signals for the triply, respectively five-fold charged species ([**A2**@**C1**_2_ + H]^3+^ at *m*/*z* = 729 and [**A4**@**C1**_4_ + H]^5+^ at *m*/*z* = 719) could be observed. The most abundant species – most probably due to its high ESI response factor – is the one sodium ion containing molecular ion of **C1** ([Na@**C1**]^+^ at *m*/*z* 471, see Supporting Information File 1). The spectra of the di- and tetravalent hosts **C2** and **C4** and the monovalent guest **A1** show a more complex signal pattern (Figure 6c,d). In the mixture of divalent crown ether **C2** with **A1** three different species in a statistical distribution of 1:2:1 were detected: the host with two axles [**A1**_2_@**C2**]^2+^ (*m*/*z* = 948), the host with one axle and one sodium ion [Na**A1**@**C2**]^2+^ (*m*/*z* = 861) and the host loaded with two sodium ions ([Na_2_@**C2**]^2+^ *m*/*z* = 773). This can be easily explained with the nature of the ESI spray process, which is known to cause the dissociation in multiply charged non-covalently bound complexes. The results of the NMR titrations, however, clearly indicate the doubly bound pseudorotaxane **A1**_2_@**C2** to be the most prominent species in solution (Figure 4a). The fact that the desired pseudorotaxane **A1**_2_@**C2** can be detected by mass spectrometry despite the likely dissociation of the multiply charged complex in the ion source shows that this technique gives reasonable results for determining the species present in solution. The 4:1 mixture of **A1** and **C4** gives rise to an even more complex signal pattern (Figure 6d). Due to the four binding sites of **C4**, there are numerous possibilities of **A1** and sodium cations to bind. There are species with three or four guest ions detected with an approximately statistic distribution: [Na_(4−_*_x_*_)_**A1***_x_*@**C4**]^4+^ (*x* = 1, 2, 3, 4) and [Na_(3−_*_y_*_)_**A1***_y_*@**C4**]^3+^ (*y* = 1, 2, 3). The desired [5]pseudorotaxane is not very stable at the ionization conditions, but is nevertheless detected ([**A1**_4_@**C4**]^4+^ at *m*/*z* = 737). As explained above, this shows that mass spectrometry gives a reasonable image of the species present in solution, because we already know from NMR titration studies that the [5]pseudorotaxane **A1**_4_@**C4** is the predominant species in solution (Figure 4b).

To summarize, all four desired [3]- or [5]pseudorotaxanes could be detected by mass spectrometry despite the likeliness of **A1**_2_@**C2** and **A1**_4_@**C4** to dissociate upon electrospray ionization. These results show that mass spectrometry should be a well suited method for the investigation of the multivalent pseudorotaxanes under study. These usually show much higher binding constants than the monovalent analogue and should therefore very likely survive the ionization process.

### [2]- and [3]pseudorotaxanes from di- and tetravalent building blocks

**Figure 7:**
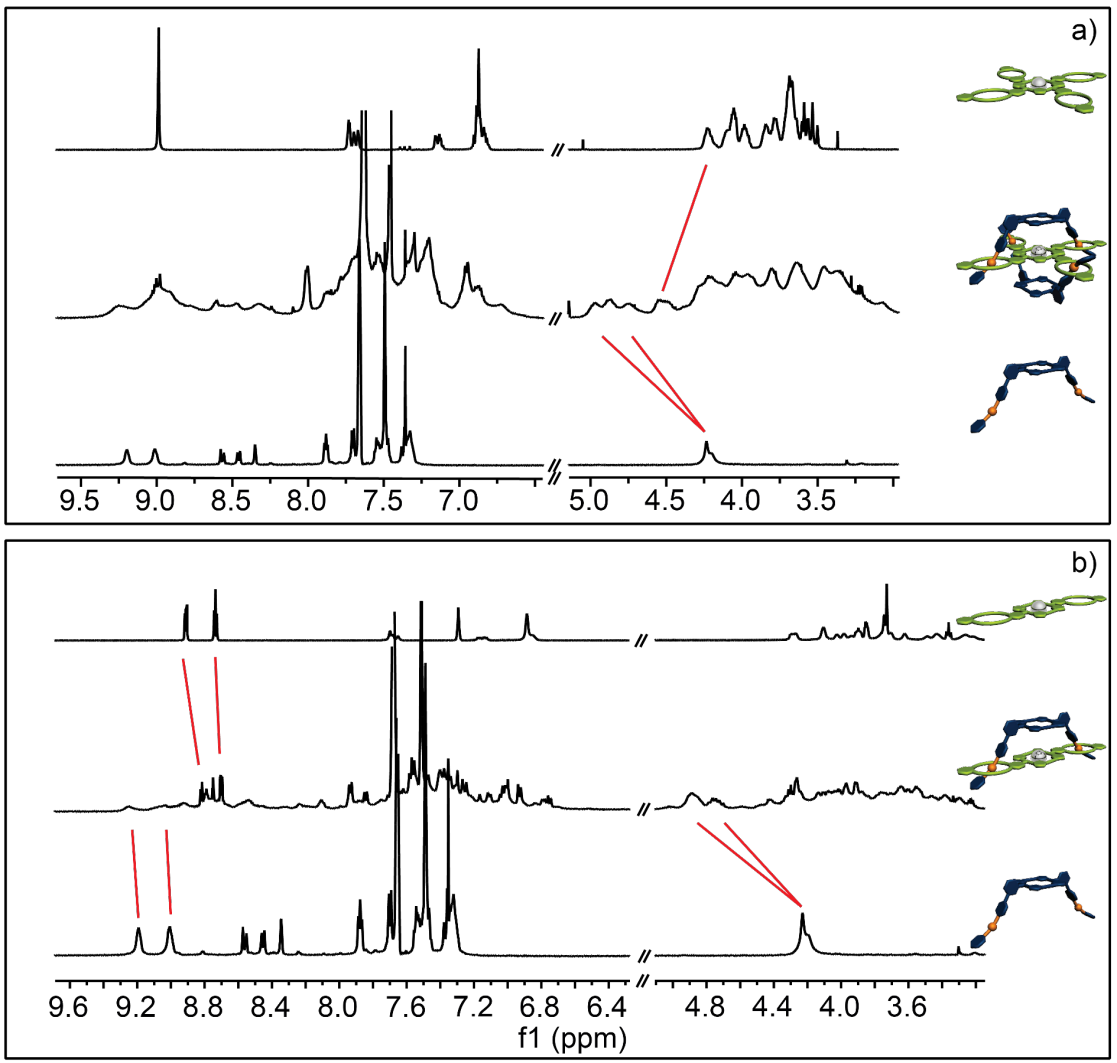
^1^H NMR (500 MHz, 298 K, CD_2_Cl_2_, 1 mM) of a) **C4** (top), **A2_2_**@**C4** (middle) and **A2** (bottom); b) **C2** (top), **A2**@**C2** (middle) and **A2** (bottom). Disappearance and shift of the signals (red lines) suggest complexation. Due to the presence of a complex stereoisomeric mixture only qualitative information of the complexation is possible.

**Figure 8:**
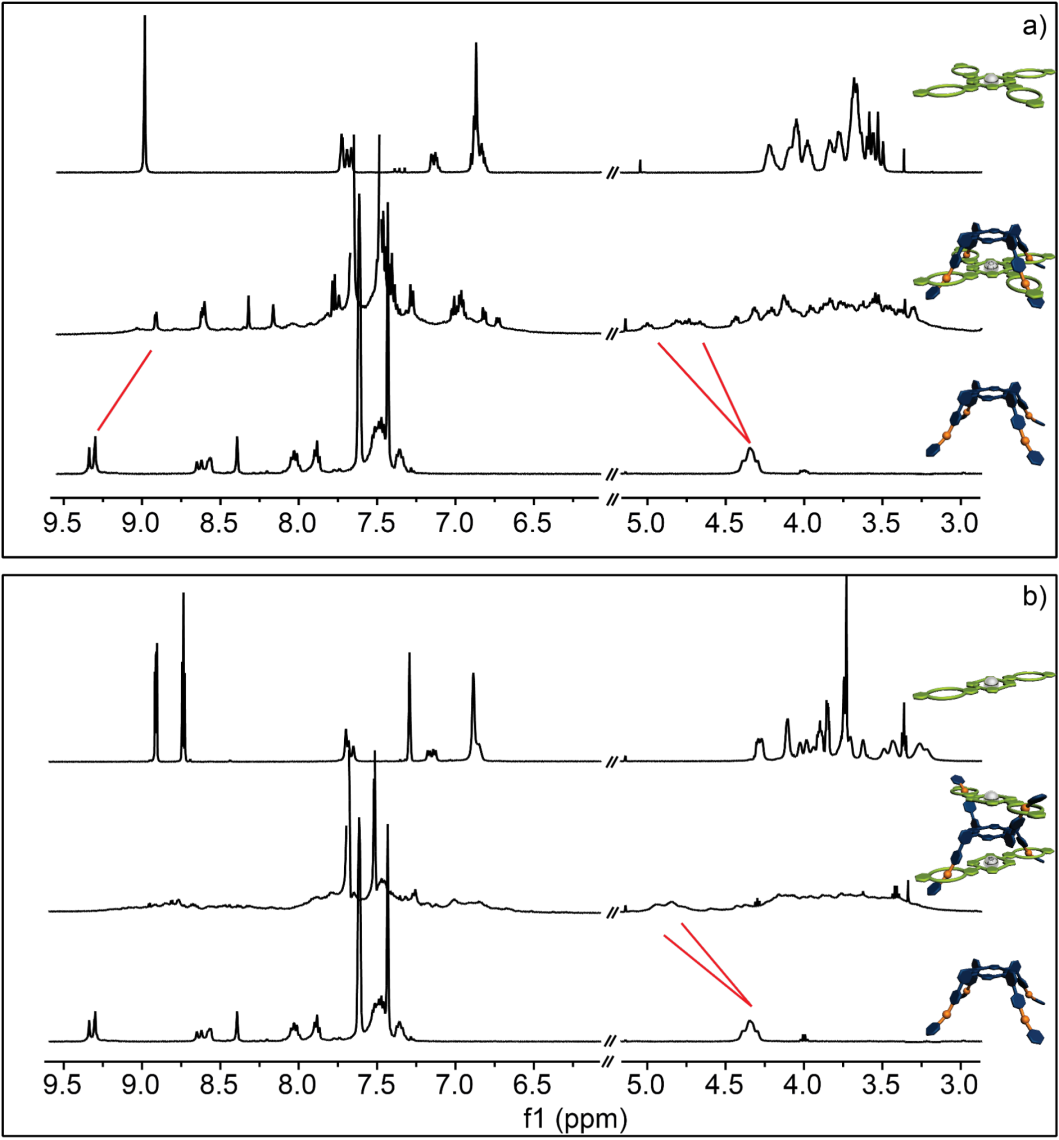
^1^H NMR (500 MHz, 298 K, CD_2_Cl_2_, 1 mM) of a) **C4** (top), **A4**@**C4** (middle) and **A4** (bottom) and b) **C2** (top), **A4**@**C2_2_** (middle) and **A4** (bottom). Disappearance and shift of the signals (red lines) suggest complexation. Due to the presence of a complex stereoisomeric mixture only qualitative information of the complexation is possible.

Subsequently, we investigated the di- and tetravalent pseudorotaxanes formed between **A2**, **A4**, **C2**, and **C4**. As already mentioned above, NMR spectroscopy is limited for the given systems because of the numerous isomers that can be formed. However, some general conclusion can be made. In all four cases one can observe a shift of the benzylic protons H^b/c^ down field by approximately 0.5 ppm, which is typical for the threading in a crown ether/secondary ammonium ion binding motif. Furthermore, the signals for the crown ether region broaden significantly, which is in agreement with the assumption that upon complexation the number of signals increases because the methylene protons become diasterotopic and different supramolecular stereoisomers can form. However, based on the present NMR spectroscopy data (Figure 7 and Figure 8) one cannot exclude the formation of polymeric aggregates or only partially threaded structures. For this reason the formed complexes were analyzed in detail using mass spectrometry.

**Figure 9:**
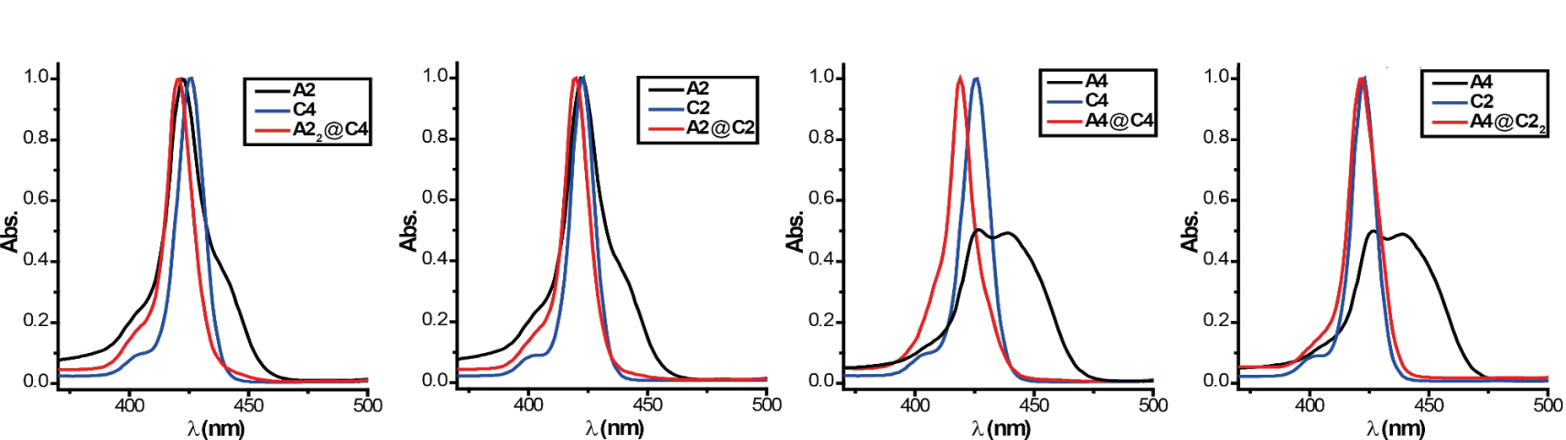
Normalized UV–vis absorption spectra (CH_2_Cl_2_, 2 μM) of the guests **A2** and **A4** (black), the hosts **C2** and **C4** (blue) and of the mixtures (red), showing a slight hypsochromic shift of the absorption maxima upon complexation.

Comparing the absorption of the complexes (Figure 9), one can see that the tetravalent **A4**@**C4** complex shows the strongest blue shift while the divalent **A4**@**C2_2_** shows almost no change in the spectrum (except breaking the **A4** aggregate). The hypsochromic shift indicates a parallel alignment of the porphyrin moieties, which is in good agreement with the hypothesized structure. However, since the observed shifts are rather small the interactions, i.e., exciton coupling, between the two porphyrin chromophores seems to be rather weak.

**Figure 10:**
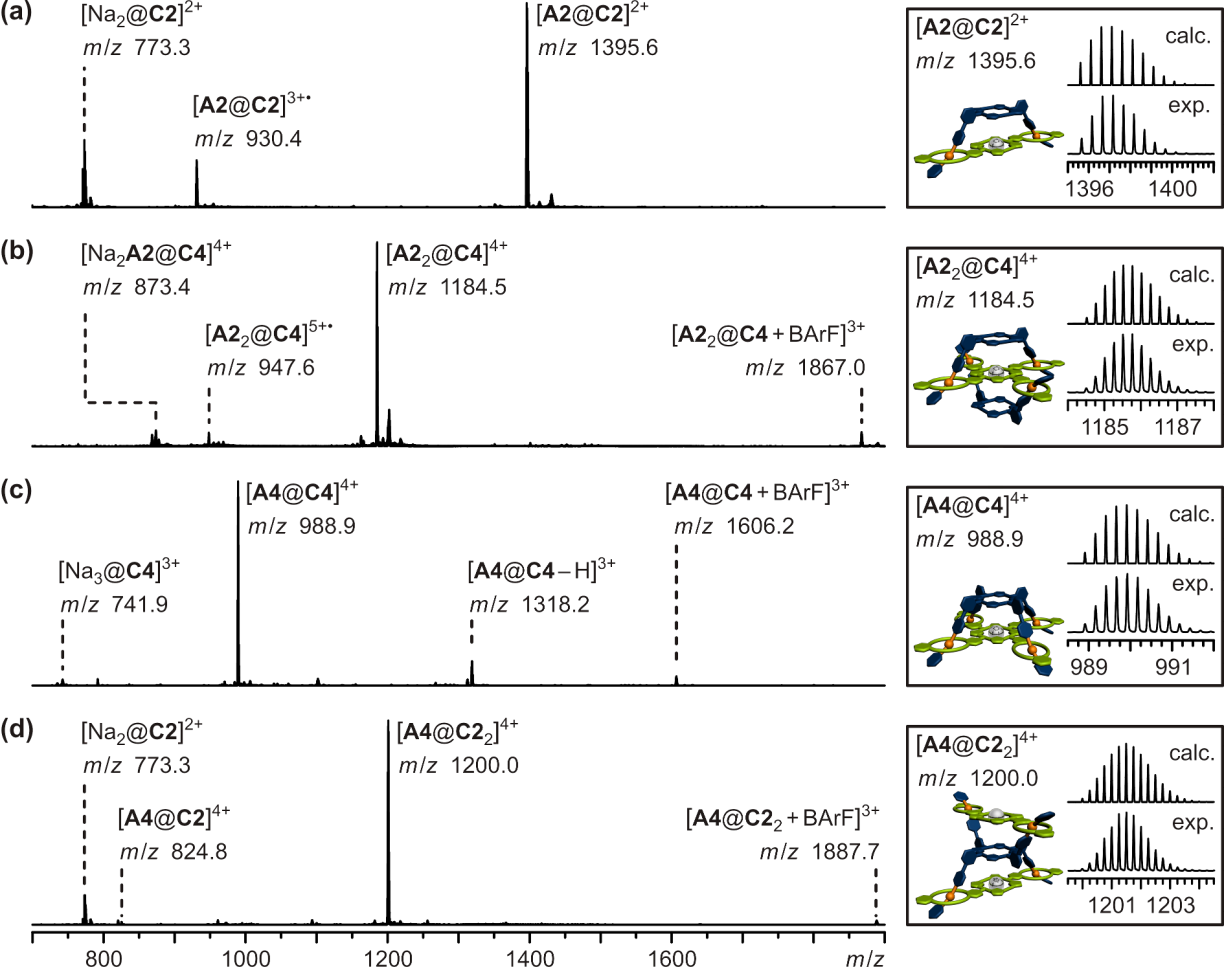
ESI-Q-TOF-MS spectra (CH_2_Cl_2_, 0.2 µM; left hand side) and respective experimental and calculated isotopic patterns of the desired [2]- or [3]pseudorotaxanes (right hand side): a) 1:1 mixture of **A2** and **C2**, b) 2:1 mixture of **A2** and **C4**, c) 1:1 mixture of **A4** and **C4**, d) 1:2 mixture of **A4** and **C2**.

For mass spectrometric analysis (ESI-Q-TOF MS) of the desired pseudorotaxanes separate solutions of hosts and guests were prepared (CH_2_Cl_2_, **A2**/**C2**: 0.6 mM, **A4**/**C4**: 0.3 mM). They were mixed in the respective 1:1, 1:2, and 2:1 molar ratios and allowed to equilibrate for 14 hours at 6 °C, after which no further changes in the mass spectra were observed and thus equilibrium was reached. The pseudorotaxane solutions were diluted to 0.2 µM prior to analysis. The respective mass spectra are shown in Figure 10. Guest **A2** was combined with host **C2** as well as **C4** in 1:1 and 2:1 ratios, respectively. The expected pseudorotaxanes [**A2**@**C2**]^2+^ (*m*/*z* = 1396) and [**A2**_2_@**C4**]^4+^ (*m*/*z* = 1185) are detected as the major species (Figure 10a,b). A species with only one guest **A2** in host **C4** [Na_2_**A2**@**C4**]^4+^ (*m*/*z* = 873) could also be detected but with very low intensity. This partly bound species **A2**@**C4** could in principle allow formation of small oligomers, if present in solution. The fact that no oligomers could be detected and the very small abundance of the signal of the partly bound state [Na_2_**A2**@**C4**]^4+^ (*m*/*z* = 873) leads to the conclusion, that this partly bound pseudorotaxane is most probably a product of the electrospray ionization process.

In cases of the 1:1 mixture of **A4** and **C4** and the 1:2 mixture of **A4** and **C2** the desired pseudorotaxanes [**A4**@**C4**]^4+^ (*m*/*z* = 989) and [**A4**@**C2**_2_]^4+^ (*m*/*z* = 1200) are the most abundant species and there are again only traces of the possible 1:1 pseudorotaxane [**A4**@**C2**]^4+^ (*m*/*z* = 825) detected (Figure 10c,d). As mentioned above, this is most probably a product of the ionization process. The free hosts **C4** and **C2** are detected in only small amounts or traces. Again, in both cases no oligomers are observed.

In summary, the formation of all desired multivalent pseudorotaxanes of building blocks **A2**, **A4**, **C2**, and **C4** could be verified by mass spectrometry. The defined stoichiometry for the observed pseudorotaxanes in the gas phase ([**A2**@**C2**]^2+^, [**A2**_2_@**C4**]^4+^, [**A4**@**C4**]^4+^, [**A4**@**C2**_2_]^4+^), the only slight abundance of partly bound pseudorotaxanes ([Na_2_**A2**@**C4**]^4+^, [**A4**@**C2**]^4+^) and the absence of any oligomeric species gives clear evidence, that this specific binding situation is also present in solution.

## Conclusion

The successful synthesis of di- and tetravalent porphyrin-based guests **A2** and **A4** as well as their complementary di- and tetravalent hosts **C2** and **C4** could be achieved. All four molecules show strong binding even to simple monovalent building blocks **A1** and **C1**, respectively, which could be shown by NMR-titration experiments as well as mass spectrometry. Furthermore, the formation of the di- and tetravalent pseudorotaxanes **A2**@**C2**, **A2_2_**@**C4**, **A4**@**C2_2_**, and **A4**@**C4** could be demonstrated qualitatively by NMR spectroscopy and was investigated in detail by mass spectrometry. Since the association constants in the monovalent cases are already too high to be determined by NMR-titration experiments, currently ongoing work is dealing with the daunting task to quantify the binding constants for the di- and tetravalent multiporphyrin complexes for example using isothermal calorimetry (ITC), in order to analyze the thermodynamics and kinetics of multivalent binding in these architectures in detail. In the future, we will continue to exploit the concept of complementary multivalent binding to program the increasingly complex self-assembly of multiple different chromophore components into functional supramolecular architectures.

## Supporting Information

File 1 Detailed synthetic procedures.
