# Supplementary material for: Discrete multiporphyrin pseudorotaxane assemblies from di- and tetravalent porphyrin building blocks
Source: Beilstein J Org Chem. 2015 May 12;11:748–62. doi: 10.3762/bjoc.11.85 (PMC4464431; doi:10.3762/bjoc.11.85)
Supplement: File 1 — Detailed synthetic procedures. [file Beilstein_J_Org_Chem-11-748-s001.pdf]

Supporting Information

for

Discrete multiporphyrin pseudorotaxane assemblies from di- and tetravalent porphyrin building blocks

Mirko Lohse[1], Larissa K. S. von Krbek[2], Sebastian Radunz[1], Suresh Moorthy,[2] Christoph A. Schalley*[,2] and Stefan Hecht*[,1]

Address: [1]Department of Chemistry, Humboldt-Universität zu Berlin, Brook-Taylor-Str. 2, 12489 Berlin, Germany. Fax: +49 (0)30 2093-6940; Tel: +49 (0)30 2093-7308 and [2] Institut für Chemie und Biochemie, Freie Universität Berlin, Takustraße 3, 14195 Berlin, Germany. Fax: +49(0)308385-5366; Tel: +49(0)308385-2639

Email: Stefan Hecht - sh@chemie.hu-berlin.de; Christoph A. Schalley - christoph@schalley-lab.de

Detailed synthetic procedures

General Methods S2

Synthetic procedures and compound characterization data S3

^{1}H NMR titrations of the pseudorotaxanes S18

Mass spectrometric analysis S20

References S23

General methods

Solvents and commercial starting materials were used as supplied. The solvents were dried before use, if necessary, employing an Innovative Technologies solvent purification system (multi-unit micro series). Silica gel for chromatography from Merck (0.035–0.070 mm, 60 Å) was used for column chromatography. The petroleum ether (PE) used had a boiling range of 40–60 °C. NMR spectra were recorded on a 500 MHz (125 MHz for ^{13}C) Bruker AVANCE II 500 spectrometer or on a 300 MHz (75.6 MHz for ^{13}C) Bruker DPX 300 spectrometer at 25 °C using the solvent residual proton signals as internal standard (^{1}H: δ($CHCl_3$) = 7.26 ppm, δ(CH_2Cl_2) = 5.32 ppm, δ(CH_3CN) = 1.94 ppm, ^{13}C: δ($CHCl_3$) = 77.16 ppm, ^{13}C: δ(CH_2Cl_2) = 53.5 ppm, ^{13}C: δ(CH_3CN) = 118.3 and 1.32 ppm). Ultrahigh-performance liquid chromatography connected to mass spectrometry (UPLC/MS) was performed on a Waters Acquity UPLC equivuipped with a Waters LCT Premier XE Mass detector for high resolution MS (HRMS, ESI^+ ionization) and with Waters Alliance systems (consisting of a Waters Separations Module 2695, a Waters Diode Array Detector 996 and a Waters Mass Detector ZQ 2000). TLC was performed on Merck Silica Gel 60 F254 TLC plates with a fluorescent indicator employing a 254 nm UV lamp for visualization. All procedures linked to photochemistry were performed using spectrophotometric grade solvents.

UV–vis spectroscopy was performed on either a Varian Cary 50 or Varian Cary 60 UV–vis spectrophotometer equivuipped with a Peltier thermostated cell holder at 25 ± 0.05 °C.

Electrospray ionization reflectron-time-of-flight high resolution mass spectrometric (ESI-ReTOF-HRMS) experiments were conducted on an Agilent 6210 ESI-TOF,

Agilent Technologies, Santa Clara, CA, USA. The flow rate was set to 4 µL/min and the spray voltage to 4 kV. The desolvation gas was set to 1 psi (1 bar). All other parameters were optimized for maximum abundance of the respective $[M + H]^+$.

Electrospray ionization quadrupole-time-of-flight high resolution mass spectrometric (ESI-Q-TOF-HRMS) experiments were performed with a Synapt G2-S HDMS, Waters Co., Milford, MA, USA. The flow rate was set to 10 µL/min and the spray voltage to 3 kV (1.5 kV for **A1**$_4$@**C4**). The desolvation gas was set to 1 psi (1 bar). All other parameters were optimized for maximum abundance of the respective $[M]^+$.

Compounds NaBArF [1], **C1** [2], **6** [3], **18** [4] were synthesized according to literature.

Synthetic procedures and compound characterization data

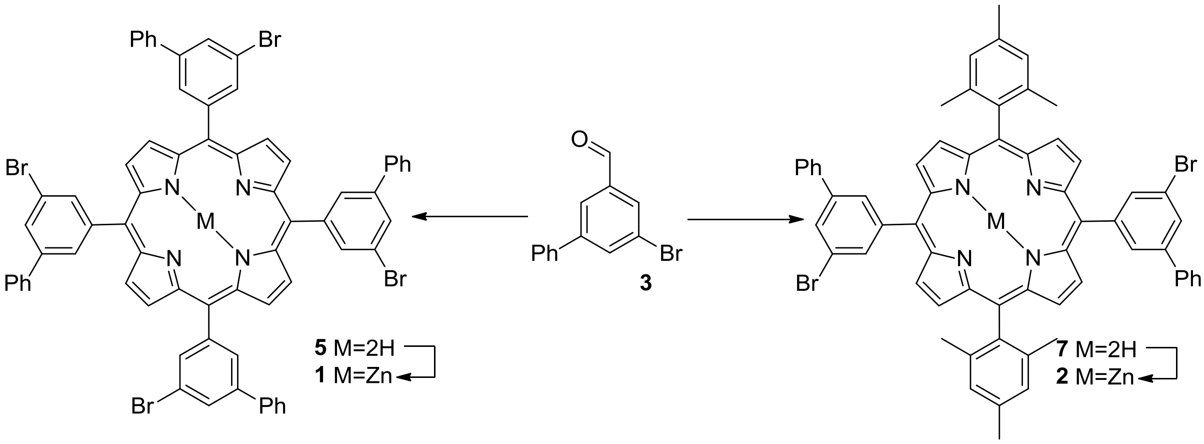

3-Bromo-5-phenyl-benzaldehyde (3)

The reactant 3,5-dibromobenzaldehyde (6.44 g, 24.40 mmol, 1 equiv) was added to a mixture of phenylboronic acid (2.41 g, 21.96 mmol, 0.9 equiv), $Pd(PPh_3)_4$ (70 mg, 1.22 mmol, 0.05 equiv), 2 M aqueous Na_2CO_3 solution (20 mL), ethanol (40 mL) and toluene (120 mL). The reaction mixture was stirred at 90–95 °C under a nitrogen atmosphere overnight. After cooling to room temperature the organic layer was separated and the aqueous layer was extracted with Et_2O (3 x 50 mL). The combined

organic phases were dried over $MgSO_4$ and evaporated to give a crude solid. Purification by column chromatography afforded **5** as a colorless oil (4.787 g, 18.30 mmol, yield 75%). 3,5-Diphenylbenzaldehyde (400 mg) was isolated as the major side-product.

^{1}H NMR (500 MHz, $CDCl_3$): δ = 10.01 (s, CHO), 8.01 (m, $1H_{aryl}$), 7.96 (m, $2H_{aryl}$), 7.59 (m, $2H_{aryl}$), 7.49 (m, $2H_{aryl}$), 7.43 (m, $1H_{aryl}$) ppm **^{13}C NMR** (125 MHz, $CDCl_3$): δ = 190.6, 144.0, 138.2, 138.2, 135.6, 130.9, 129.1, 128.6, 127.0, 126.8, 123.7 ppm. **ESI-MS:** could not be ionized by the ESI-source.

5,10,15,20-Tetrakis{(3-bromo-5-phenyl)-phenyl}porphyrin (5)

Pyrrole (**4**, 0.207 mL, 3.00 mmol, 1 equiv), compound **3** (783 mg, 3.00 mmol, 1 equiv) were dissolved in $CHCl_3$ (400 mL) and 0.6 g molecular sieves (6 Å) were added. The mixture was degassed by bubbling argon through the stirred solution for 15 min after which $BF_3{\cdot}Et_2O$ (0.040 mL, 0.3 mmol, 0.1 equiv) was added dropwise and stirring was continued for 4 h. Afterwards DDQ (680 mg, 3.0 mmol, 1 equiv) was added and stirring was continued for a further hour, followed by the addition of TEA (2 mL). The major impurities were removed by filtration of the crude reaction mixture through a silca plug with CH_2Cl_2 as the eluting solvent. Pure product **5** was obtained by subsequent column chromatography (SiO_2, petroleum ether/CH_2Cl_2 7:3) as shimmering violet crystals (270 mg, 0.88 mmol, 29%).

^{1}H NMR (500 MHz, $CDCl_3$): δ = 9.01 (s, $8H_{aryl}$), 8.42 (m, $8H_{aryl}$), 8.23 (t, J = 1.70 Hz, $4H_{aryl}$), 7.84 (m, $8H_{aryl}$), 7.52 (m, 8H, H_{aryl}), 7.43 (m, $4H_{aryl}$), -2.78 (s, 2H, NH) **^{13}C NMR** (125.75 MHz, CD_2Cl_2): δ = 144.1, 141.4,139.4, 139.3, 135.9, 132.2, 132.2, 129.7, 129.1, 128.2, 127.5, 121.6, 118.9 ppm.

Zinc[5,10,15,20-tetrakis{(3-bromo-5-phenyl)phenyl}porphyrin] (1)

$Zn(OAc)_2$ (461 mg, 2.10 mmol, 10 equiv) was added to a stirring solution of **5** (259 mg, 0.21 mmol, 1 equiv) in 30 mL of a chloroform/methanol solvent and the reaction mixture was stirred at room temperature overnight. After removal of the solvents under reduced pressure, the crude product was redissolved in CH_2Cl_2 and washed with brine. The separated organic layer was dried over $MgSO_4$ and concentrated. Purification by column chromatography (SiO_2, petroleum ether/CH_2Cl_2 7:3) afforded **1** as violet crystals (270 mg, 2.09 mmol, 99%).

^{1}H NMR (500 MHz, $CDCl_3$): δ = 9.01 (s, 8H_{aryl}), 8.45 (s, 8H, H_{aryl}), 8.22 (s, 4H_{aryl}), 7.84 (m, 8H_{aryl}), 7.47 (m, 8H_{aryl}), 7.39 (m, 4H_{aryl}) **^{13}C NMR** (125.75 MHz, $CDCl_3$): δ = 150.1, 144.8, 141.2, 139.4, 135.9, 132.3, 132.1, 129.4, 129.0, 128.1, 127.4, 121.1, 119.8 ppm. **ESI-MS:** 1298.0 (calc. 1298.0 $C_{68}H_{40}N_4Br_4Zn$).

5,15-Bis{(3-bromo-5-phenyl)phenyl}-10,20-(bismesityl)porphyrin (7)

Molecular sieves (6 Å, 0.06 g) were added to a stirring solution of 5-bromo-[1,1'biphenyl]-3-carbaldehyde (**3**, 0.227 g, 0.87 mmol, 1 equiv) and 2,2'-(mesitylmethylene)bis(1*H*-pyrrole) (**6**, 0.230 g, 0.87 mmol, 1 equiv) in 300 mL of chloroform. The mixture was degassed by bubbling argon through the solution for 20 min before $BF_3\cdot Et_2O$ (0.03 mL, 0.190 mmol, 0.2 equiv) were added. The reaction mixture was stirred in the dark for 2 h, after which DDQ (0.197 g, 0.87 mmol, 1.0 equiv) was added and the stirring was continued overnight. Afterwards TEA (1 mL) was added and the major impurities were removed by filtration of the crude reaction mixture through a silica plug with CH_2Cl_2 as the eluting solvent. Pure product **7** was obtained by column chromatography (SiO_2, CH_2Cl_2/petroleum ether 1:3) as a purple solid (0.120 g, 0.24 mmol, 28%).

^{1}H NMR (500 MHz, $CDCl_3$): δ = 8.87 (d, J = 4.8 Hz, $4H_{aryl}$), 8.74 (d, J = 4.8 Hz, $4H_{aryl}$), 8.45 - 8.32 (m, $4H_{aryl}$), 8.18 (s, $4H_{aryl}$), 7.87 - 7.75 (m, $4H_{aryl}$), 7.45 (m, $8H_{aryl}$), 7.28 (m, $4H_{aryl}$), 2.62 (s, 6H), 1.85 (s, 12H), -2.64 (s, 2H, NH) **^{13}C NMR** (125.75 MHz, $CDCl_3$): δ =144.4, 141.3, 139.5, 139.4, 139.3, 138.2, 137.9, 135.8, 132.0, 129.6, 129.1, 128.2, 127.9, 127.5, 121.5, 118.9, 117.6, 21.7, 21.5 ppm.

Zinc[5,15-bis{(3-bromo-5-phenyl)phenyl}-10,20-(bismesityl)porphyrin] (2)

Zinc(II) acetate (0.131 g, 0.60 mmol, 5 equiv) was added to a stirring solution of **7** (0.120 g, 0.12 mmol, 1 equiv) in 20 mL of chloroform/methanol 1:1 and stirred overnight at room temperature. Afterwards the solvent was removed under reduced pressure and the crude product was purified by column chromatography (SiO_2, PE/CH_2Cl_2 3:1) to obtain **2** as a purple/red solid (0.110 g, 0.10 mmol, 86%).

^{1}H NMR (500 MHz, $CDCl_3$): δ = 8.98 (d, J = 4.6 Hz, $4H_{aryl}$), 8.80 (d, J = 4.6 Hz, $4H_{aryl}$), 8.52 - 8.32 (m, $4H_{aryl}$), 8.20 (t, J = 1.7 Hz, $2H_{aryl}$), 7.83 (d, J = 7.5 Hz, $2H_{aryl}$), 7.49 (t, J = 7.8 Hz, $2H_{aryl}$), 7.41 (m, $4H_{aryl}$), 7.30 (s, $4H_{aryl}$), 2.62 (s, 6H), 1.82 (s, 12H). **^{13}C NMR** (125.76 MHz, $CDCl_3$): δ =150.2, 149.8, 145.2, 139.7, 139.3, 138.7, 137.6, 135.8, 132.2, 131.2, 129.1, 128.2, 127.8, 127.5, 120.0, 118.5, 21.7, 21.5 ppm. **ESI-MS:** 1072.3 (calc. 1072.3 $C_{62}H_{46}Br_2N_4Zn$)

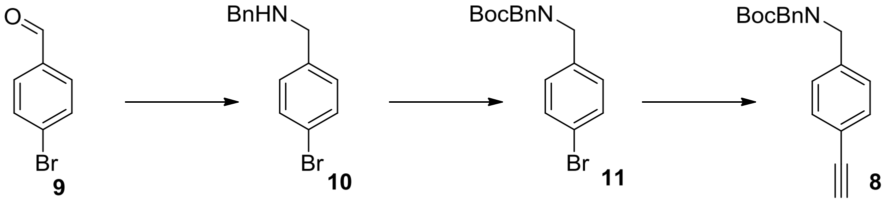

Benzyl-(4-bromobenzyl)amine (10)

Benzylamine (1.158 g, 10.81 mmol, 1 equiv) was added to a solution of 4-bromo-benzaldehyde (**9**, 2.000 g, 10.81 mmol, 1 equiv) in 15 mL of trimethyl orthoformate and stirred overnight. The solvent was removed under reduced pressure to obtain the

desired product in decent purity which was used without further purification (2.91 g, 10.80 mmol, 98%). $NaBH_4$ (0.817 g, 21.59 mmol, 2 equiv) were added to an ice-cooled solution of the imine (2.960 g, 10.80 mmol, 1 equiv) in a 1:1 mixture of THF/MeOH (30 mL each). The mixture was allowed to warm to room temperature and stirred for 2 h. The volatiles were removed under reduced pressure and the crude product was dissolved in ethyl acetate. The slurry mixture was filtered through a celite plug using ethyl acetate as the solvent. Pure product **10** was obtained after removal of the solvent under reduced pressure (2.910 g, 10.79 mmol, 98%).

^{1}H NMR (500 MHz, $CDCl_3$): δ = 7.38(m, 2H_{aryl}), 7.26(m, 2H_{aryl}), 7.15(m, 5H_{aryl}), 3.72(s, 2H), 3.69 (s, 2H) **^{13}C NMR** (125.75 MHz, CDCl3): δ = 155.9, 131.7, 128.5, 27.4, 80.3, 28.5 ppm. **ESI-HRMS** 276.019 (calc. 276.021 $C_{14}H_{15}BrN^+$)

(*tert*-Butyloxycarbonyl)(benzyl)4-bromobenzylamine (11)

Triethylamine (2 mL) was added to a stirred solution of **10** (2,688 g, 9.77 mmol, 1 equiv) and $(Boc)_2O$ (3.271 g, 15.00 mmol, 1.5 equiv) dissolved in 100 mL CH_2Cl_2. After all of the starting material was consumed (TLC, ca 16 h) the solvent was removed under reduced pressure and excess $(Boc)_2O$ was removed by bulb-to-bulb distillation. The crude product was purified by column chromatography (SiO_2, petroleum ether/EtOAc 99:1 → 4:1) affording pure **11** as a colorless oil (2.200 g, 5.87 mmol, 60%).

^{1}H NMR (500 MHz, $CDCl_3$): δ = 7.45 (m 2H_{aryl}), 7.23 (m,7H_{aryl}), 4.34 (br, 4H), 1.49 (s, 9H) **^{13}C NMR** (125.75 MHz, $CDCl_3$): δ = 140.1, 139.3, 131.5, 129.9, 128.5, 128.2, 127.1, 120.7, 53.1, 52.4 ppm. **ESI-HRMS**: 320.056 (calc. 320.040 $C_{15}H_{15}BrNO_2^+$)

(*tert*-Butyloxycarbonyl)(benzyl){4-(trimethylsilyl)ethynylbenzyl}amine (12)

CuI (5 mg, 0.03 mmol, 0.1 equiv), triphenylphosphine (35 mg, 0.14 mmol, 0.5 equiv), and **11** (100 mg, 0.27 mmol, 1 equiv) were dissolved in 4 mL of a 1:1 mixture of dry toluene/triethylamine which was degassed by rapid vacuum–argon cycles (5 times). Afterwards, $Pd(PPh_3)_4$ (31 mg, 0.03 mmol, 0.1 equiv) was added and the solution was degassed again (3 times). After the addition of ethynyltrimethylsilane (0.08 mL, 0.53 mmol, 2 equiv) the solution was stirred at 80 °C for 16 h. When all starting material was consumed (TLC) the mixture was filtered through a plug of celite with ethyl acetate as the eluting solvent. The resulting solution was concentrated under reduced pressure and purified by column chromatography (SiO_2, petroleum ether/EtOAc 15:1 → 9:1) to yield **12** as a colourless solid (0.100 g, 0.26 mmol, 96%).

^{1}H NMR (500 MHz, $CDCl_3$): δ = 7.45 (m, 2H_{aryl}), 7.34 (m, 7H_{aryl}), 4.38 (br, 4H), 1.50 (s, 9H), 0.28 (s, 9H) **^{13}C NMR** (125.75 MHz, $CDCl_3$): δ = 155.9, 132.1, 128.5, 128.0, 127.8, 127.4, 127.3, 127.1, 104.9, 94.1, 80.2, 49.3, 28.4, 0.4 ppm. **ESI-HRMS**: 338.158 (calc. 338.157 $C_{20}H_{24}NO_2Si^+$)

(*tert*-Butyloxycarbonyl)(benzyl)4-ethynylbenzylamine (8)

Potassium hydroxide (36 mg, 0.64 mmol, 2.5 equiv) was added to a solution of **12** (100 mg, 0.25 mmol, 1 equiv) in toluene and stirred overnight at room temperature. Afterwards the solvent was removed under reduced pressure and the residue was dissolved in CH_2Cl_2, washed sequentially with 1 M HCl and brine, dried over $MgSO_4$ and concentrated under reduced pressure. The crude product was purified by column chromatography (SiO_2, petroleum ether:EtOAc 9:1) to give an yellowish oil that solidifies upon standing (65 mg, 0.23 mmol, 90%).

^{1}H NMR (500 MHz, $CDCl_3$): δ = 7.51 (m, 2H_{aryl}), 7.33 (m,7H_{aryl}), 4.42 (br, 4H), 3.17 (s, 1H), 1.51 (s, 9H) **^{13}C NMR** (125.75 MHz, $CDCl_3$): δ = 156.1, 132.4, 128.7, 128.2,

128.0, 127.5, 121.1, 80.4, 77.3, 28.6 ppm. **ESI-HRMS**: 338.128 (calc. 338.157 $C_{17}H_{16}NO_2^+$)

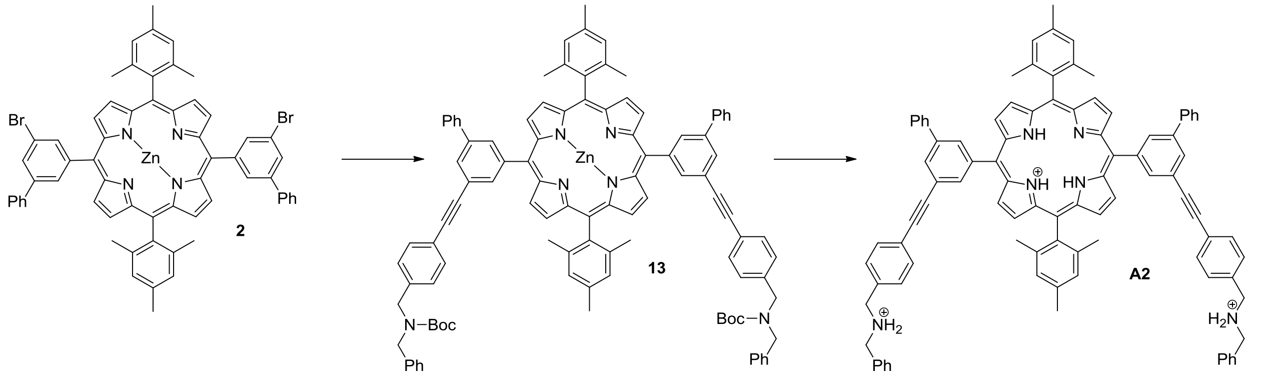

Boc-protected bisammonium precursor porphyrin (13)

CuI (1.7 mg, 0.01 mmol, 0.2 equiv), triphenylphosphine (2.4 mg, 0.01 mmol, 0.2 equiv), and **2** (50 mg, 0.05 mmol, 1 equiv) were dissolved in 2 mL of dry toluene/triethylamine 1:1 and degassed by rapid vacuum–argon cycles (5 times). Afterwards, $Pd(PPh_3)_4$ (11 mg, 0.09 mmol, 0.2 equiv) was added and the solution was degassed again (3 times). After the addition of **8** (75 mg, 0.23 mmol, 5 equiv), the solution was stirred at 80 °C for 3 d under a nitrogen atmosphere. When all starting material was consumed (TLC), the mixture was filtered through a plug of celite with CH_2Cl_2 as the solvent. The resulting solution was concentrated under reduced pressure and purified by column chromatography (SiO_2, CH_2Cl_2). The product was precipitated from CH_2Cl_2/MeOH by slowly removing CH_2Cl_2 under vacuum (72 mg, 0.03 mmol, 69%).

^{1}H NMR (600 MHz, $CDCl_3$): δ = 9.02 (m, 4H_{aryl}), 3.93 (m, 4H_{aryl}), 8.45 (br, 4H_{aryl}), 8.25 (s, 2H_{aryl}), 7.56 (m, 8H_{aryl}), 7.35 (m, 20H_{aryl}), 4.20 (br, 8H), 2.66 (s, 6H), 1.89 (s, 12H), 1.32 (br, 18H) **^{13}C NMR** (125.76 MHz, $CDCl_3$): δ = 155.7, 145.0, 149.8, 143.9, 140.2, 139.4, 139.3, 139.2, 137.7, 137.3, 135.9, 133.1, 132.1, 131.8, 130.8, 129.2, 128.9,

128.9, 128.0, 127.8, 127.6, 127.5, 127.3, 121.8, 119.2, 118.7, 80.1, 65.7, 28.3, 24.1, 21.7 ppm.

Target bisammonium porphyrin salt (A2)

Trifluoroacetic acid (3 mL) was added to a stirring solution of **13** (150 mg, 0.10 mmol, 1 equiv) in CH_2Cl_2 and the resulting solution was stirred for 3 d. When no starting material was left (TLC), the solvent was removed under reduced pressure until dryness, the residue was dissolved in CH_2Cl_2 and washed with 1 M NaOH solution. The combined organic phases were dried over $MgSO_4$ and concentrated under reduced pressure to obtain **14** as a purple solid (120 mg, 0.10 mmol, 97%) which was used without further purification.

^{1}H NMR (300 MHz, MeOD): δ = 9.03 (d, J = 4.9 Hz, 4H_{aryl}), 8.83 (d, J = 4.9 Hz, 4H_{aryl}), 8.72 (m ,2H_{aryl}), 8.61 (m, 2H_{aryl}), 8.41 (m, 2H_{aryl}), 7.96 (d, J = 7.8 Hz, 4H_{aryl}), 7.76 (d, J = 8.2 Hz, 4H_{aryl}), 7.50 (m, 28H_{aryl}), 4.29 (s, 4H), 4.28 (s, 4H), 2.65 (s, 6H), 1.95 (s, 12H) ppm.

To a solution of **14** (120 mg, 0.10 mmol, 1 equiv) in 20 mL chloroform/methanol 1:1 was added 3 mL of 1 M HCl solution. After stirring for 5 min the solution was concentrated to dryness under reduced pressure to obtain a green solid. This process was repeated three times to fully protonate the compound. The hydrochloride of **14** (100 mg, 0.07 mmol, 1 equiv) was then dissolved in methanol and NaBArF (196 mg, 0.22 mmol, 3.1 equiv) was added. The desired product was precipitated by adding water to the solution and subsequent centrifugation. The obtained sticky solid was dissolved in MeOH, again precipitated with water and centrifuged. This process was repeated three times. **A2** was obtained as a purple solid after lyophilisation from benzene (0.160 mg, 0.02 mmol, 58%).

^{1}H NMR (600 MHz, CD_2Cl_2): δ = 9.20 (s, $4H_{aryl}$), 9.01 (s, $4H_{aryl}$), 8.56 (m, $2H_{aryl}$), 8.45 (m, $2H_{aryl}$), 8.34 (s, $2H_{aryl}$), 7.88 (m, $4H_{aryl}$), 7.70 (m, $4H_{aryl}$), 7.66 (m, $24H_{aryl}$), 7.54 (m, $16H_{aryl}$), 7.49 (s, $8H_{aryl}$), 7.33 (m, $12H_{aryl}$), 4.23 (br, 8H), 2.63 (s, 6H), 1.81 (s, 12H) **^{13}C NMR** (125.76 MHz, MeOD): δ =163.7, 163.3, 162.4, 135.8, 133.5, 131.3, 131.0, 130.9, 130.6, 130.4, 130.4-130.3 , 129.1 - 128.9, 128.5, 126.9, 124.7, 118.5, 52.3, 51.8, 49.9 **^{19}F NMR** (470 MHz, MeOD) -63.85 ppm. **ESI-HRMS**: 645.317 (calc. 645.313)

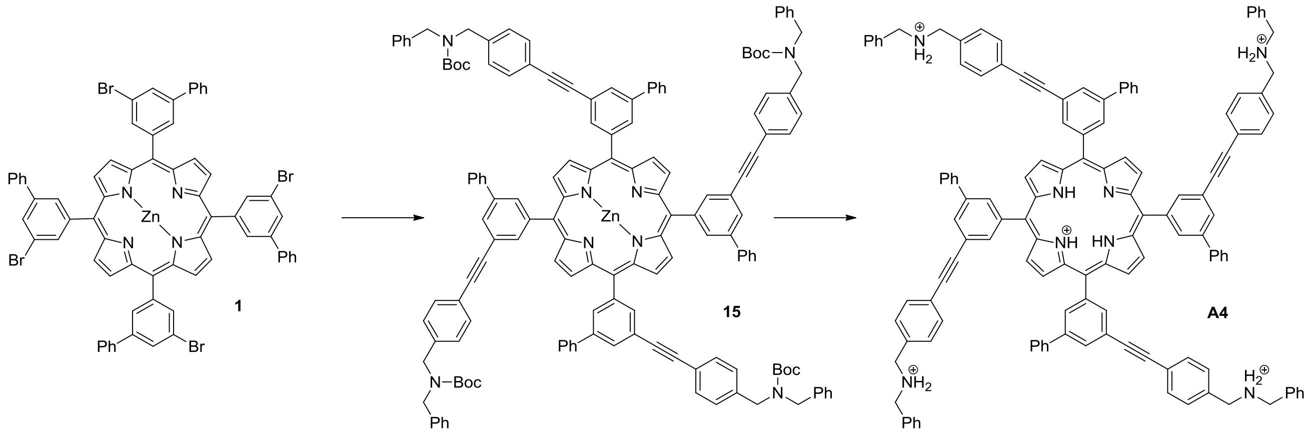

Boc-protected tetraammonium precursor porphyrin (15)

Porphyrin **1** (260 mg, 0.20 mmol, 1.0 equiv), PPh_3 (21 mg, 0.08 mmol, 0.4 equiv) and CuI (15 mg, 0.08 mmol, 0.4 equiv) were dissolved in 40 mL toluene/TEA 1:1 and degassed by rapid vacuum–argon cycles (5 times). Afterwards, $Pd(PPh_3)_4$ (92 mg, 0.08 mmol, 0.4 equiv) was added and the flask was degassed again (3 times). After **8** (643 mg, 2.00 mmol, 10.0 equiv) was added the solution was stirred at 80 °C for 3 d under a nitrogen atmosphere. Purification was conducted on a short silica plug with CH_2Cl_2 as the eluent, followed by column chromatography (silica gel, CH_2Cl_2/MeOH 100:1) and finally precipitated from (MeOH/CH_2Cl_2) by slowly removing the CH_2Cl_2 under vacuum to obtain pure **15** (421 mg, 0.19 mmol, 93%) as a purple solid.

^{1}H NMR (500 MHz, $CDCl_3$): δ = 9.12 (s, 8H_{aryl}), 8.47 (s, 4H_{aryl}), 8.40 (s, 4H_{aryl}), 8.24 (s, 4H_{aryl}), 7.89 (m, 8H_{aryl}), 7.55 (m, 8H_{aryl}), 7.52 (m, 8H_{aryl}), 7.42 (m, 4H_{aryl}), 7.31 (m, 8H_{aryl}), 7.26 (m, 4H_{aryl}), 7.13 (m, 16H_{aryl}), 4.28 (m, 16H), 1.40(s, 36H) **^{13}C NMR** (125.76 MHz, $CDCl_3$): δ = 156.7, 150.3, 143.6, 140.1, 139.6, 137.7, 136.1, 133.4, 132.3, 131.9, 129.4, 129.0, 128.6, 128.0, 127.9, 127.5, 127.3, 122.1, 120.2, 89.7, 80.2, 49.2, 28.3 ppm.

Target tetraammonium porphyrin salt (A4)

Precursor **15** (396 mg, 0.18 mmol, 1.0 equiv) was dissolved in 20 mL of CH_2Cl_2 and 6 mL of TFA was added. After stirring overnight at room temperature the reaction mixture was concentrated under reduced pressure. The crude solid was redissolved in CH_2Cl_2 and washed with 2 M aqueous $NaHCO_3$ solution. The combined organic phases were dried over $MgSO_4$ and the solvent was removed under reduced pressure to obtain the desired compound **16** (310 mg, 0.18 mmol, 98%) as a purple solid that was used without further purification.

Compound **16** (314 mg, 0.18 mmol, 1.0 equiv) was dissolved in 40 mL of a 1:1 $CHCl_3$/MeOH mixture and 3 mL 1 M aqueous HCl were added. After 5 min of stirring the volatiles were removed under reduced pressure and this procedure was subsequently repeated another 3 times. The so obtained solid was dried under vacuum overnight. The above hydrochloride of **16** (0.335 g, 0.17 mmol, 1 equiv) was dissolved in 20 mL MeOH and NaBArF (825 mg, 0.93 mmol, 5.5 equiv) was added. After 10 min of stirring, H_2O was added slowly until the precipitation of the desired compound occurs. The precipate was collected by centrifugation and was then redissolved in MeOH to repeat the precipitation. This procedure was repeated two more times to obtain **A4** (703 mg, 0.12 mmol, 68%).

^{1}H NMR (500 MHz, CD_2Cl_2): δ = 9.30 (s, $8H_{aryl}$), 8.60 (s, $8H_{aryl}$), 8.39 (s, $4H_{aryl}$), 8.03 (m, $8H_{aryl}$), 7.88 (m, $8H_{aryl}$), 7.61 (s, $40H_{aryl}$), 7.43 (s, $20H_{aryl}$), 7.54-7.34 (m, $40H_{aryl}$), 4.28 (m, 16H) **^{13}C NMR** (126 MHz, MeOD) δ 163.5, 163.1, 162.7, 162.3, 135.8, 133.5, 132.2, 131.3, 131.0, 130.9, 130.6, 130.4, 130.3, 130.1, 129.4, 129.0, 128.5, 126.9, 125.7, 124.7, 122.5, 118.5, 91.4, 90.3, 52.2, 51.7 **^{19}F NMR** (470 MHz, MeOD) -64.40 ppm

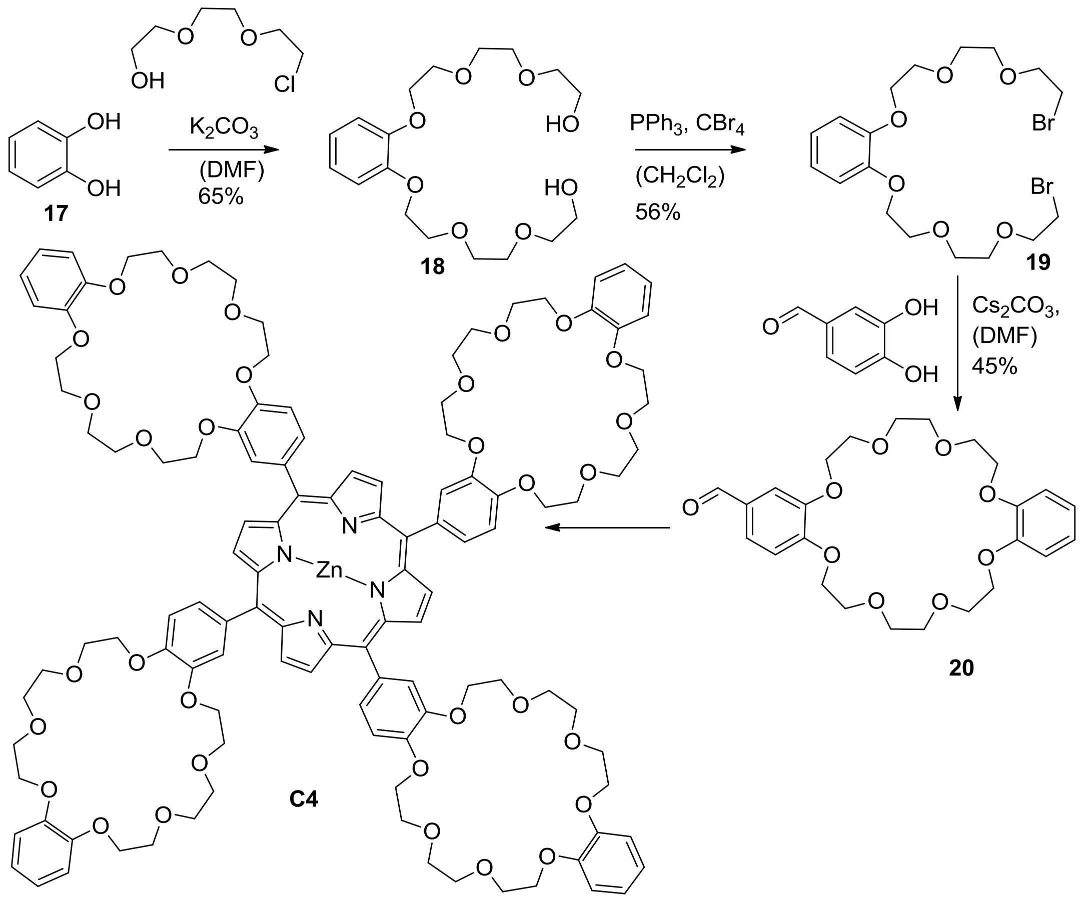

1,2-Bis(2-(2-(2-bromoethoxy)ethoxy)ethoxy)benzene (19)

PPh_3 (56.71 g, 261.40 mmol, 6 equiv) followed by CBr_4 (71.710 g, 261.40 mmol, 6 equiv) were added to a stirring solution of **18** (13.494 g, 36.04 mmol, 1 equiv) in CH_2Cl_2. The mixture was stirred overnight at room temperature, concentrated under reduced pressure and purified by column chromoatography (SiO_2, CH_2Cl_2/PE 9:1) to obtain **19** as a yellow oil (10.34 g, 20.54 mmol, 57%).

^{1}H NMR (300 MHz, $CDCl_3$): δ = 6.92 (s, $4H_{aryl}$), 4.17 (m, 4H), 3.87 (m, 4H), 3.81 (m, 4H), 3.72 (m, 8H), 3.47 (m, 4H) **^{13}C NMR** (126 MHz, $CDCl_3$) δ = 148.6, 121.3, 114.6, 70.8, 70.4, 70.2, 69.5, 68.6, 30.2 ppm. **ESI-HRMS**: 499.030 (calc. 499.025 $C_{18}H_{28}O_6Br_2^+$)

3-Formyl-dibenzo[24]crown-8 (20)

3,4-Dihydroxybenzaldehyde (1.381 g, 10.00 mmol, 1 equiv) was dissolved in 100 mL of dry DMF in two necked round-bottomed flask and Cs_2CO_3 powder (11.40 g, 35.00 mmol, 3.5 equiv) was added. The mixture was allowed to stir for 15 min, after which **19** (5.50 g, 12.66 mmol, 1.1 equiv) dissolved in 100 mL of dry DMF was added dropwise over 2 h at 60 °C. Then the temperature was raised to 85 °C and the reaction mixture was stirred for 5 d at this temperature. Afterwards the solvent was removed under reduced pressure, 100 mL of water were added to the residue and the mixture was extracted with CH_2Cl_2 three times. The combined organic extracts were dried over anhydrous sodium sulfate and the solvent was removed under reduced pressure to give the crude product. Subsequent purification by column chromatography (SiO_2, CH_2Cl_2/MeOH 98:2) yielded **20** (2.1 g, 4.00 mmol, 40%) as a white powder.

^{1}H NMR (300 MHz, $CDCl_3$): δ = 9.79 (s, 1H), 7.40 (dd, J = 8.2, 1.9 Hz, $1H_{aryl}$), 7.35 (d, J = 1.9 Hz, $1H_{aryl}$), 6.92 (dd, J = 8.5, 3.5 Hz, $1H_{aryl}$), 6.88 - 6.81 (m, $4H_{aryl}$), 4.19 (m, 4H), 4.15 - 4.09 (m, 4H), 3.96 - 3.85 (m, 8H), 3.82 (d, J = 3.8 Hz, 8H) **^{13}C NMR** (126 MHz, $CDCl_3$) δ = 190.8, 162.4, 154.2, 151.6, 149.0, 148.7, 130.0, 126.7, 121.6, 113.8, 111.8, 110.9, 72.5, 71.4, 71.3, 71.1, 70.3, 69.8, 69.3. ppm. **ESI-HRMS**: 499.030 (calc. 494.238 $C_{25}H_{32}O_9NH_4^+$)

5,10,15,20-Tetrakis (dibenzo[24]-crown-8)porphyrin (21)

Pyrrole (0.1 mL, 1.50 mmol, 1 equiv) was added to a hot solution (100 °C) of **20** (720 mg, 1.50 mmol, 1 equiv) in 115 mL of propionic acid and the solution was refluxed for 3 h. After removal of the solvent by distillation, the black crude product was purified by column chromatography (neutral alumina, CH_2Cl_2) and preparative GPC to obtain **21** as a purple solid (100 mg, 0.19 mmol, 13%).

^{1}H NMR (300 MHz, $CDCl_3$): δ =9.02 (s, 8H_{aryl}), 7.74 (m, 8H_{aryl}), 7.24 - 7.16 (m, 8H_{aryl}), 6.98 - 6.81 (m, 16H_{aryl}), 4.29 (m, 16H), 4.20 - 3.99 (m, 32H), 3.96 - 3.81 (m, 16H), 3.81 - 3.69 (m, 32H) **^{13}C NMR** (125.75 MHz, $CDCl_3$): δ = 172.1, 171.5, 170.1, 149.0, 148.8, 147.0, 153.3, 128.0, 121.5, 120.6, 119.8, 114.16, 111.88, 104.9, 96.9, 82.0, 80.2, 77.3, 71.6, 71.5, 71.4, 70.19, 70.13, 70.08, 70.06, 69.7, 69.6, 69.5, 69.4, 29.8, 14.6, 1.1 ppm

Zinc [5,10,15,20-tetrakis(dibenzo[24]-crown-8)porphyrin] (C4)

Zinc acetate (21 mg, 0.10 mmol, 2 equiv) was added to a stirring solution of **21** (100 mg, 0,05 mmol, 1 equiv) in 20 mL of chloroform/methanol 1:1 and stirred at room temperature overnight. The purple solution was concentrated to dryness and purified by column chromatography (neutral alumina, CH_2Cl_2) to obtain **C4** as a purple solid (93 mg, 0.04 mmol, 90%).

^{1}H NMR (300 MHz, CD_2Cl_2): δ =9.02 (s, 8H_{aryl}), 7.74 (m, 8H_{aryl}), 7.24 - 7.17 (m, 4H_{aryl}), 6.89 (m, 16H_{aryl}), 4.25 (m, 16H), 4.10 - 3.99 (m, 24H), 3.96 - 3.85 (m, 16H), 3.81 - 3.72 (32, 4H) **^{13}C NMR** (125.75 MHz, CD_2Cl_2): δ =150.7, 149.4, 148.9, 147.7, 136.8, 132.3, 128.3, 121.7, 121.1, 114.6, 112.4, 71.3, 70.1, 70.1, 70.0 ppm**. ESI-HRMS**: 2179.81 (calc. 2179.80 $C_{116}H_{132}O_{32}N_4ZnNa^+$)

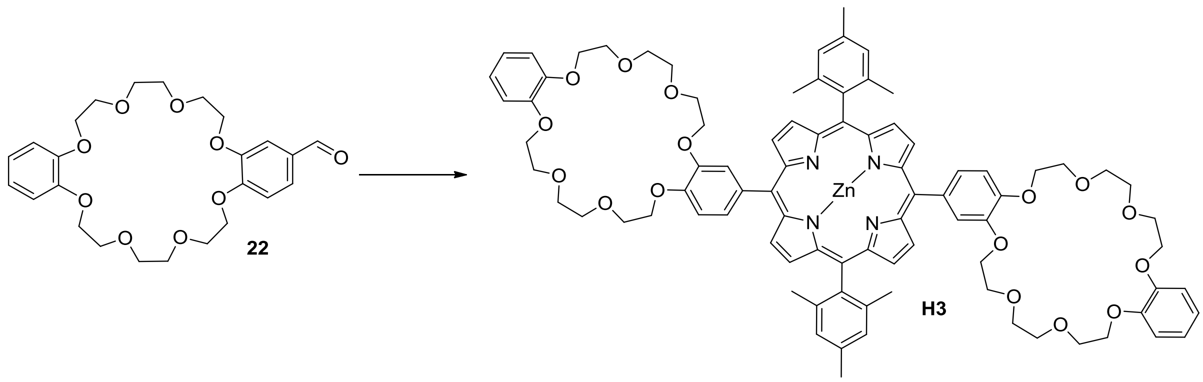

5,15-Bis(dibenzo[24]-crown-8)-10,20-bis(mesityl)porphyrin (22)

Molecular sieves (6 Å, 0.06 g) were added to a stirring solution of **20** (0.903 g, 1.90 mmol, 1 equiv), 2,2'-(mesitylmethylene)bis(1H-pyrrole), and **6** (0.501 g, 1.90 mmol, 1 equiv) in 300 mL chloroform. The mixture was degassed by bubbling argon through the solution for 20 min before $BF_3{\cdot}Et_2O$ (0.03 mL, 0.19 mmol, 0.2 equiv) was added and the mixture was stirred in the dark for 3 h at room temperature. Afterwards, DDQ (0.430 g, 1.90 mmol, 1.0 equiv) was added and stirring was continued overnight. Then TEA (1 mL) was added and the major impurities were removed by filtration through a silica plug using CH_2Cl_2 as eluent. The pure product was obtained by column chromatography (neutral alumina, CH_2Cl_2/petrolether 1:3) and preparative GPC as a purple solid (0.80 g, 1.12 mmol, 59%).

^{1}H NMR (300 MHz, CD_2Cl_2): δ = 8.90 (d, J = 4.8 Hz, 4H_{aryl}), 8.69 (d, J = 4.8 Hz, 4H_{aryl}), 7.83 (d, J = 2.0 Hz, 4H_{aryl}), 7.77 (dd, J = 8.1, 2.0 Hz, 2H_{aryl}), 7.34 (s, 4H_{aryl}), 7.29 (d, J = 8.2 Hz, 2H_{aryl}), 7.05 - 6.88 (m, 8H_{aryl}), 4.51 - 4.41 (m, 4H), 4.31 (m, 4H), 4.26 - 4.15 (m, 8H), 4.13 - 4.05 (m, 4H), 3.95 (m, 24H), 3.85 (m, 8H), 2.66 (s, 6H), 1.89 - 1.83 (m, 12H) **^{13}C NMR** (125.75 MHz, $CDCl_3$): δ = 149.0, 148.7, 147.5, 139.4, 127.8, 121.5, 114.1, 71.4, 70.0, 69.4, 21.7, 21.4 ppm.

Zinc [5,15-bis(dibenzo[24]-crown-8)-10,20-bis(mesityl)porphyrin] (C2)

Zinc acetate (229 mg, 1.04 mmol, 5 equiv) was added to a stirred solution of **22** (300 mg, 0,21 mmol, 1 equiv) in 20 mL of chloroform/methanol 1:1 and stirred overnight at room temperature. The purple solution was concentrated to dryness under reduced pressure and purified by column chromatography (neutral alumina, CH_2Cl_2) to obtain **C2** as a purple solid (298 mg, 0.20 mmol, 95%).

^{1}H NMR (500 MHz, $CDCl_3$): δ =8.81 (dd, J = 4.6, 1.8 Hz, $4H_{aryl}$), 8.68 (dd, J = 4.6, 2.5 Hz, $4H_{aryl}$), 7.69 - 7.60 (m, $4H_{aryl}$), 7.20 (s, $4H_{aryl}$), 7.06 (dd, J = 7.9, 6.5 Hz, $2H_{aryl}$), 6.85 - 6.74 (m, $8H_{aryl}$), 4.23 (m, 4H), 4.09 - 4.06 (m, 4H), 4.03 (s, 2H), 4.02 - 3.99 (m, 2H), 3.98 - 3.94 (m, 2H), 3.91 (m, 2H), 3.88 (s, 4H), 3.85 - 3.82 (m, 4H), 3.77 - 3.71 (m, 8H), 3.68 - 3.62 (m, 4H), 3.57 (s, 4H), 3.49 (s, 2H), 3.43 (s, 4H), 2.55 (s, 6H), 1.75 (s, 12H) **^{13}C NMR** (126 MHz, $CDCl_3$) δ = 150.1, 149.8, 148.8, 148.2, 148.1, 146.5, 146.4, 139.2, 137.3, 135.9, 132.3, 130.7, 127.6, 121.3, 119.7, 119.0, 113.9, 71.0, 70.9, 70.7, 70.6, 70.4, 70.3, 69.7, 69.5, 69.4, 69.3, 69.3, 69.2, 69.1, 68.9, 68.8 ppm. **ESI-HRMS**: 1523.573 (calc. 1523.570 $C_{86}H_{92}N_4O_{16}ZnNa^+$)

^{1}H NMR titrations of the pseudorotaxanes

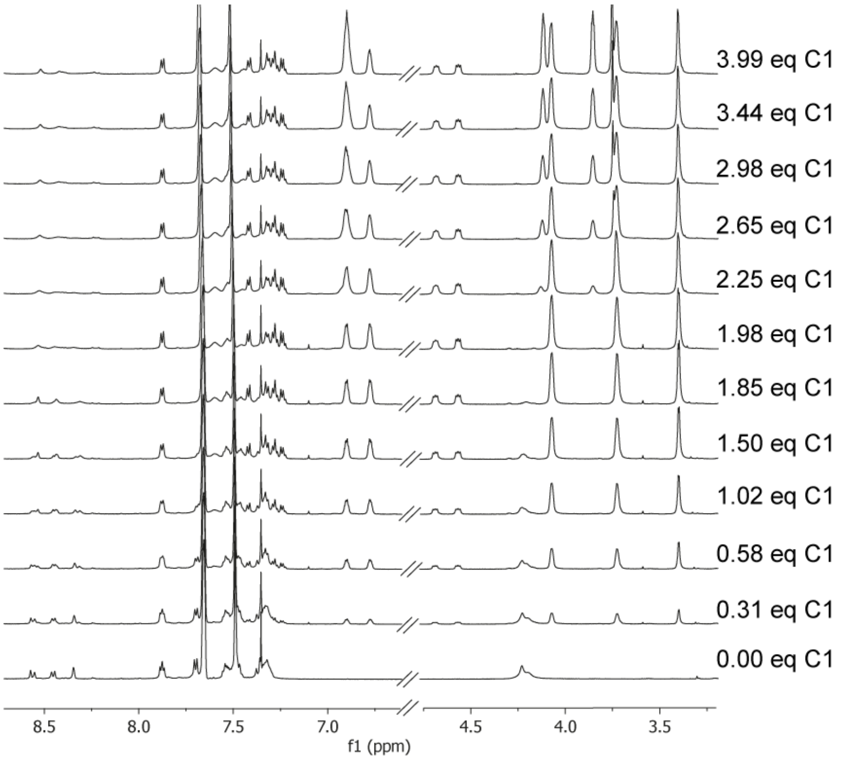

Figure S1: NMR titration (500 MHz, 298 K, CD_2Cl_2) of **C1** to a 3 mM solution of **A2** showing a slow exchange and signal shifts up to the expected 2:1 ratio.

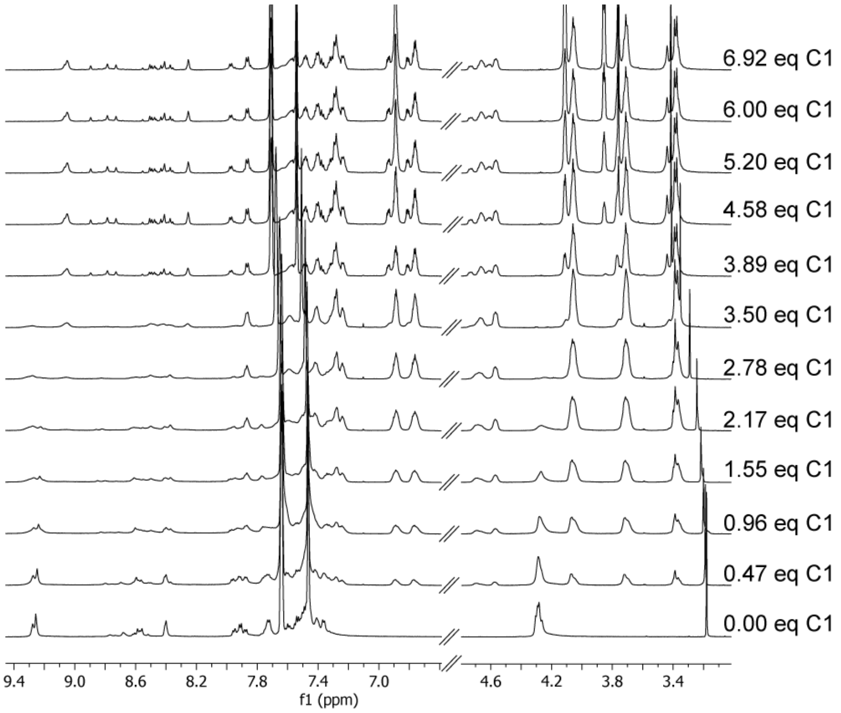

Figure S2: NMR titration (500 MHz, 298 K, CD_2Cl_2) of **C1** to a 3 mM solution of **A4** showing a slow exchange and signal shifts up to the expected 4:1 ratio.

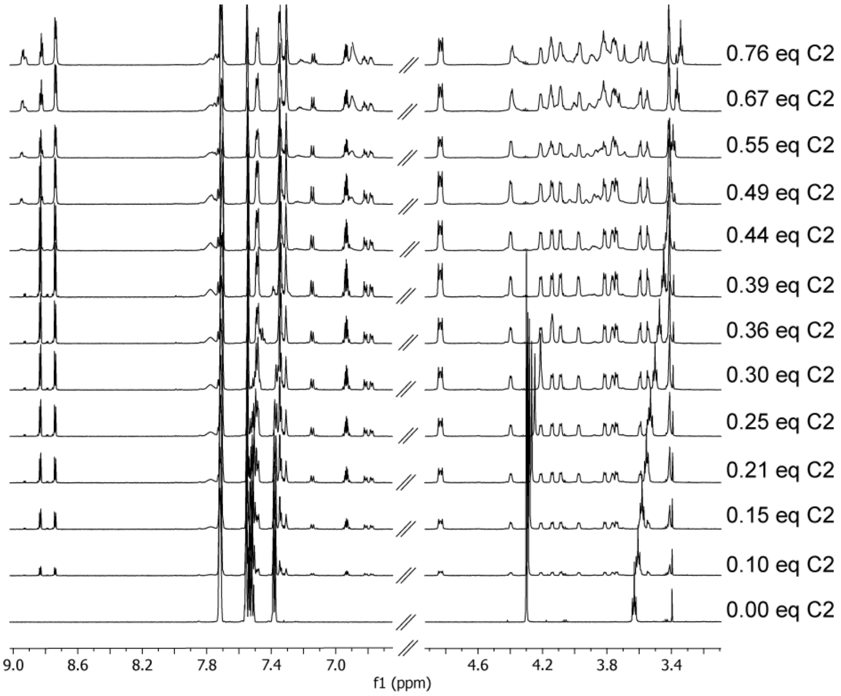

Figure S3: NMR titration (500 MHz, 298 K, CD_2Cl_2) of **C4** to a 3 mM solution of **A1** showing a slow exchange and signal shifts up to the expected 1:4 ratio.

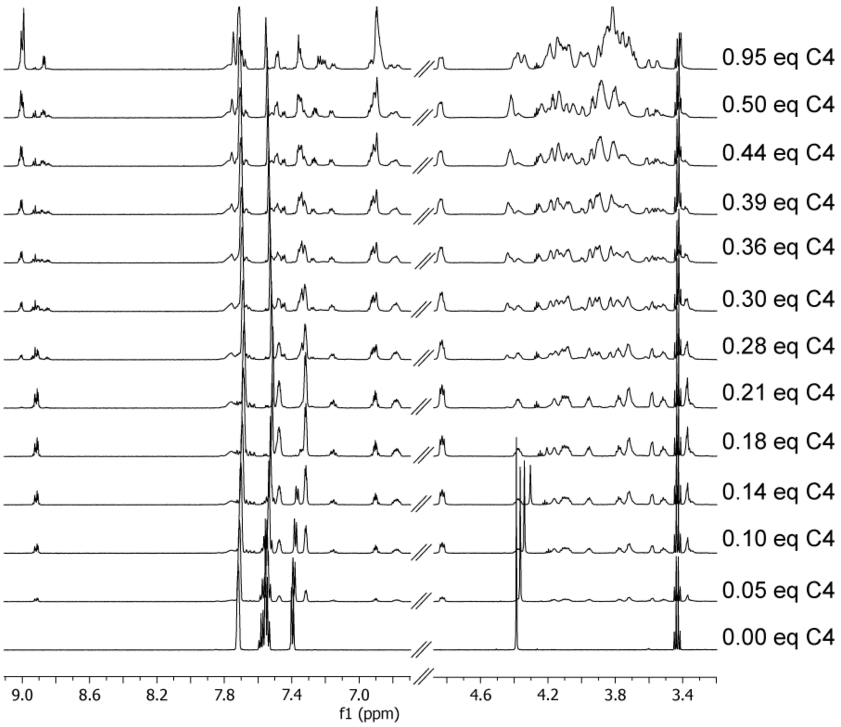

Figure S4: NMR titration (500 MHz, 298 K, CD_2Cl_2) of **C2** to a 3 mM solution of **A1** showing a slow exchange and signal shifts up to the expected 1:2 ratio.

Mass spectrometric analysis

[3]- and [5]Pseudorotaxanes with monovalent building blocks A1 or C1

The samples for mass spectrometric analysis of the [3]- and [5]pseudorotaxanes were prepared as follows. Separate solutions of hosts and guests were prepared (CH_2Cl_2, **A1**/**C1**: 4 mM, **A2**/**C2**: 2 mM, **A4**/**C4**: 1 mM), combined in the respective 1:2, 1:4, 2:1 and 4:1 molar ratios and allowed to equilibrate for 24 hours at room temperature. The solutions of the pseudorotaxanes were diluted to 0.2 µM prior to analysis.

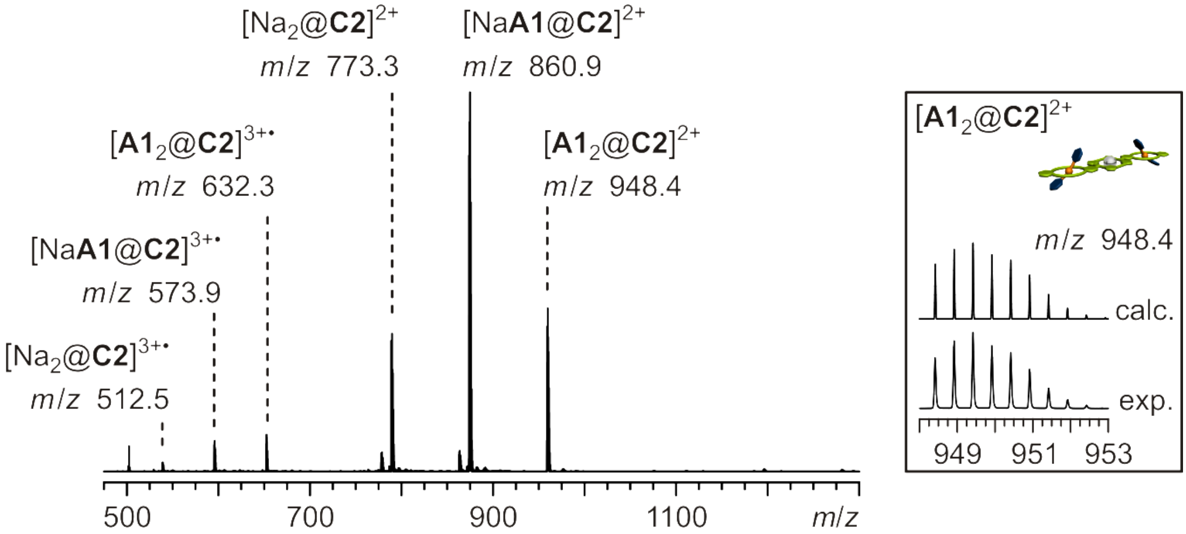

Figure S**5:** ESI-Q-TOF-MS spectrum of a 2:1 mixture of **A1** and **C2** (CH_2Cl_2, 0.2 µM; left hand side) and respective experimental and calculated isotopic patterns of the desired [3]-pseudorotaxane [$\mathbf{A1}_2$@**C2**]$^{2+}$ (*m*/*z* 948.4; right hand side).

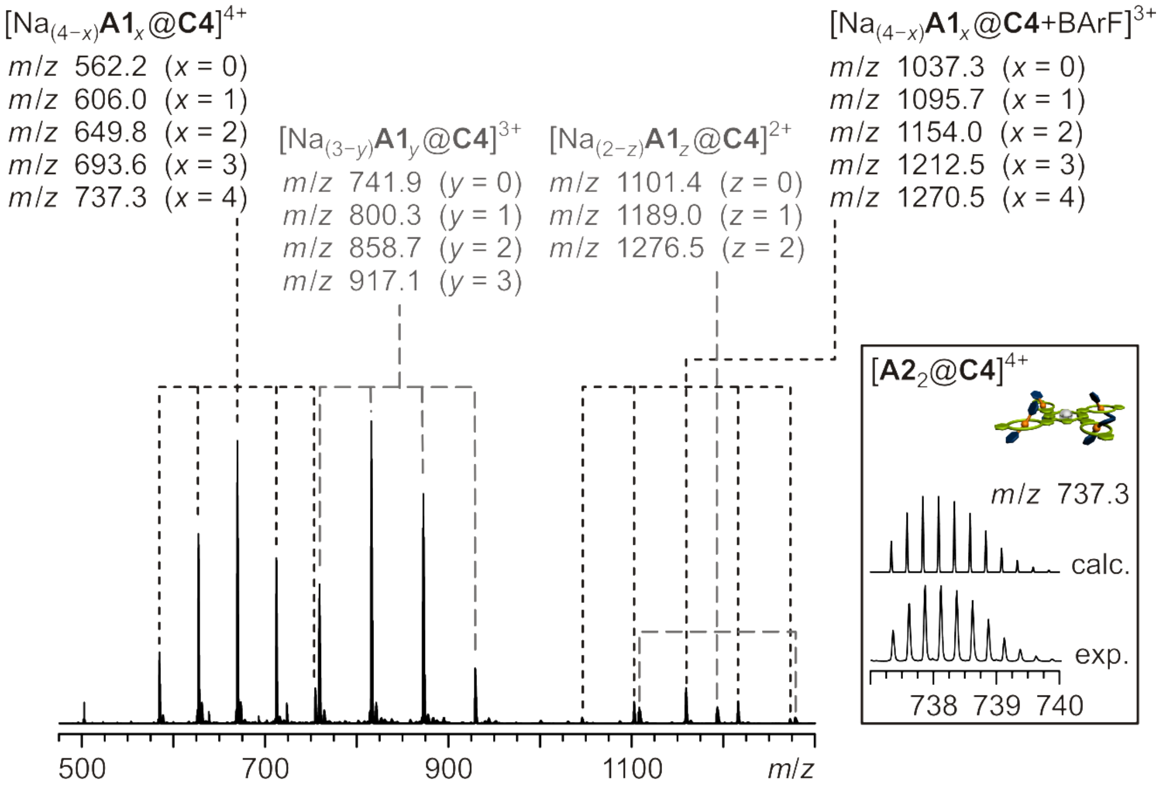

Figure S6: ESI-Q-TOF-MS spectrum of a 4:1 mixture of **A1** and **C4** (CH_2Cl_2, 0.2 µM; left hand side) and respective experimental and calculated isotopic patterns of the desired [5]-pseudorotaxane [$\mathbf{A1}_4$@**C4**]$^{2+}$ (*m*/*z* 1093.5; right hand side).

[2]- and [3]Pseudorotaxanes with di- and tetravalent building blocks A2, A4, C2 and C4

The samples for mass spectrometric analysis of the multivalent [2]- and [3]pseudorotaxanes were prepared as follows. Separate solutions of hosts and guests were prepared (CH_2Cl_2, **A2**/**C2**: 0.6 mM, **A4**/**C4**: 0.3 mM). They were mixed in the respective 1:1, 1:2 and 2:1 molar ratios and allowed to equilibrate for 14 hours at 6 °C. The pseudorotaxane solutions were diluted to 0.2 µM prior to analysis.

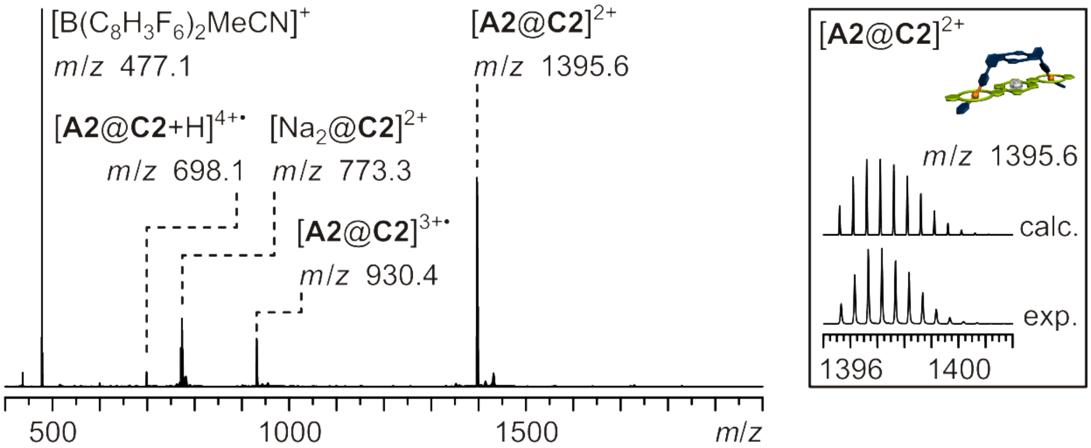

Figure S7: ESI-Q-TOF-MS spectrum of a 1:1 mixture of **A2** and **C2** (CH_2Cl_2, 0.2 µM; left hand side) and respective experimental and calculated isotopic patterns of the desired [2]pseudorotaxane [**A2@C2**]$^{2+}$ (*m/z* 1395.6033 (exp.), 1395.6039 (calc.); right hand side).

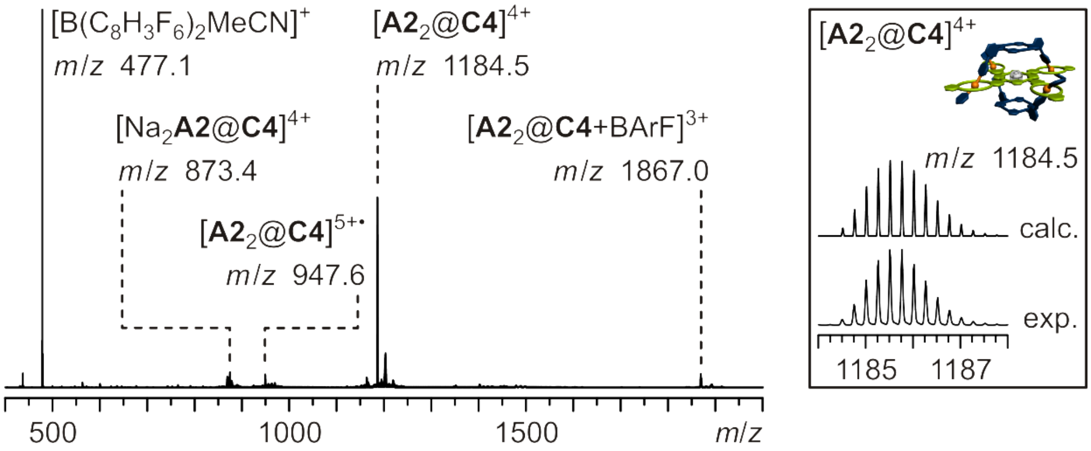

Figure S8: ESI-Q-TOF-MS spectrum of a 2:1 mixture of **A2** and **C4** (CH_2Cl_2, 0.2 µM; left hand side) and respective experimental and calculated isotopic patterns of the desired [3]-pseudorotaxane [**A2**$_2$**@C4**]$^{4+}$ (*m/z* 1184.5020 (exp.), 1184.5168 (calc.); right hand side).

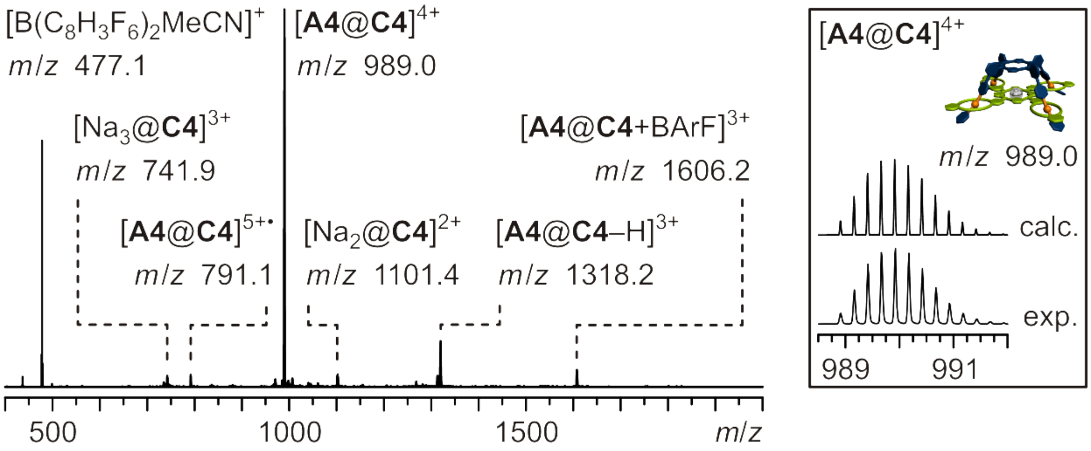

Figure S9: ESI-Q-TOF-MS spectrum of a 1:1 mixture of **A4** and **C4** (CH_2Cl_2, 0.2 µM; left hand side) and respective experimental and calculated isotopic patterns of the desired [2]-pseudorotaxane [**A4@C4**]$^{4+}$ (*m*/*z* 988.9071 (exp.), 988.9080 (calc.); right hand side).

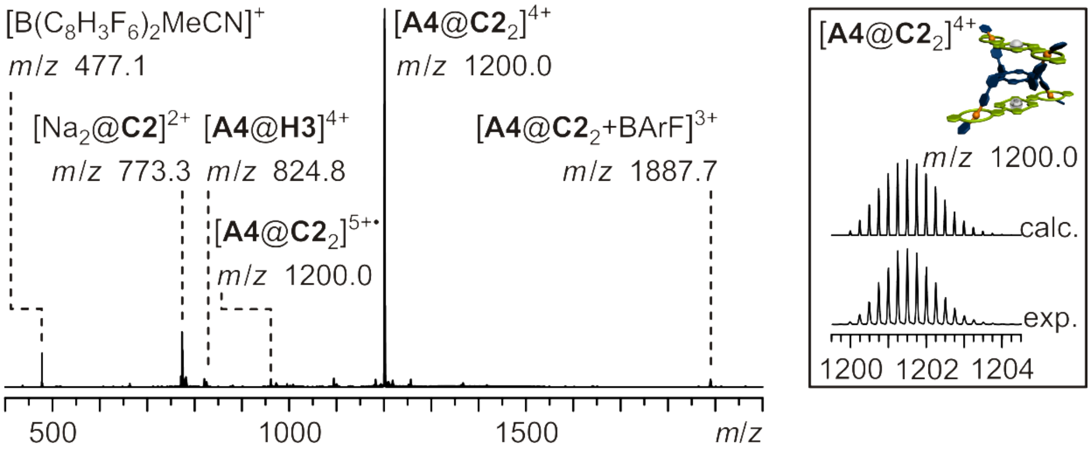

Figure S10: ESI-Q-TOF-MS spectrum of a 1:2 mixture of **A4** and **C2** (CH_2Cl_2, 0.2 µM; left hand side) and respective experimental and calculated isotopic patterns of the desired [3]-pseudorotaxane [**A4@C2**$_2$]$^{2+}$ (*m*/*z* 1199.9935 (exp.), 1199.9951 (calc.); right hand side).

References

1. D. L. Reger, T. D. Wright, C. A. Little, J. J. S. Lamba and M. D. Smith, *Inorg. Chem.*, **2001**, *40*, 3810-3814.
2. C. R. South, M. N. Higley, K. C. F. Leung, D. Lanari, A. Nelson, R. H. Grubbs, J. F. Stoddart and M. Weck, *Chem. Eur. J.*, **2006**, *12*, 3789-3797.
3. J. Frey, G. Kodis, S. D. Straight, T. A. Moore, A. L. Moore and D. Gust, *J. Phys. Chem. A*, **2012**, *117*, 607-615.

4. T. Bogaschenko, S. Basok, C. Kulygina, A. Lyapunov and N. Lukyanenko, *Synthesis*, **2002**, *2002*, 2266-2270.
